# Chemical Composition and Antifungal Activity of *Zanthoxylum armatum* Fruit Essential Oil against *Phytophthora capsici*

**DOI:** 10.3390/molecules27238636

**Published:** 2022-12-06

**Authors:** Jingjing Yang, Qizhi Wang, Linwei Li, Pirui Li, Min Yin, Shu Xu, Yu Chen, Xu Feng, Bi Wang

**Affiliations:** 1 Jiangsu Key Laboratory for the Research and Utilization of Plant Resources, Jiangsu Province Engineering Research Center of Eco-Cultivation and High-Value Utilization of Chinese Medicinal Materials, Institute of Botany, Jiangsu Province and Chinese Academy of Sciences (Nanjing Botanical Garden Mem. Sun Yat-Sen), Nanjing 210014, China; 2 Nanjing University of Chinese Medicine, Nanjing 210023, China

**Keywords:** *Phytophthora capsici*, antifungal activity, *Zanthoxylum armatum* fruit essential oil, action mechanism

## Abstract

Pathogenic plant oomycetes cause devastating damage to fruits and vegetables worldwide. Plant essential oils (EOs) are known to be promising candidates for the development of fungicides. In this study, we isolated twelve EOs from *Tetradium ruticarpum*, *Tetradium daniellii*, *Tetradium fraxinifolium*, *Zanthoxylum armatum*, *Ruta graveolens*, and *Citrus medica* leaves and fruits. We then investigated their chemical composition and antifungal activity against phytopathogenic oomycetes. Our results demonstrated that *Z. armatum* fruit essential oil (ZFO) in particular substantially inhibited the mycelial growth of *Phytophthora capsici*. Similarly, ZFO also strongly suppressed spore production and germination of *P. capsici*, and the application of ZFO significantly reduced disease symptoms caused by *P. capsici* in pepper. Furthermore, results from microscopic and biochemical studies indicated that ZFO damaged the ultrastructure and destroyed the membrane integrity of *P. capsici*, leading to the leakage of the cellular contents and ultimately causing cell death. It was concluded that ZFO could enhance the activities of defense-related enzymes in pepper fruits, which may also be responsible for the inhibition of phytophthora disease. Moreover, linalool and *D*-limonene were proven to be the primary effective components of ZFO. Our results collectively indicate that ZFO could be a potential candidate for the management of disease caused by *P. capsici*.

## 1. Introduction

Oomycete pathogens of the genera *Phytophthora*, *Pythium*, and *Plasmopara* cause considerable losses in vegetable crops worldwide [1,2]. As one of the most notorious oomycete pathogens, *Phytophthora capsici* can cause severe diseases in crops of the Cucurbitaceae, Solanaceae, and Fabaceae families. When this soil-borne disease breaks out, effective control is difficult to achieve [1,3]. To date, chemical fungicides remain the most effective measures to control this disease, due to their efficacy and ease of application. However, heavy and long-term use of chemical fungicides raises several issues including the occurrence of toxic residues and the development of pathogen resistance [2,4]. Hence, the development of effective, economic, and safe antifungal agents is urgently needed.

Natural products extracted from plants are good sources of antimicrobial compounds. Compared with chemical fungicides, they are safer and more environmentally friendly [5,6]. Plant essential oils (EOs) are volatile compounds mixtures biosynthesized in plants, mainly including terpenes, terpenoids, aliphatic, and aromatic constituents [7]. Among the various kinds of plant natural products, EOs are known to be promising candidates for the development of antifungal agents [8]. Studies reveal that plant EOs have been widely used for the disease management in various crops. For instance, Bi et al. [9] reported that some plant EOs, specifically oregano, palmarosa, and red thyme EOs, were effective for the management of *P. capsici.* Chaturvedi et al. [10] demonstrated that the EO extracted from *Psidium guajava* leaves had a significant inhibitory effect on *Curvularia lunata* and *Fusarium chlamydosporum*.

Rutaceae plants have great economic value due to their edible fruits and their EOs [11]. *Tetradium ruticarpum*, *Tetradium daniellii*, *Tetradium fraxinifolium*, *Zanthoxylum armatum*, *Ruta graveolens*, and *Citrus medica*, Rutaceae plants in the genera of *Tetradium*, *Zanthoxylum*, *Ruta*, and *Citrus*, are widely distributed in Asia, and have been cultivated in China for more than 2000 years. Among them, *Z. armatum* is used as a flavor additive and a preservative for many kinds of foods [12]. Furthermore, *Z. armatum* has been used as a traditional Chinese medicinal herb because of its functions for treating vomiting, diarrhea, ascariasis, and eczema [13]. *Z. armatum* fruit essential oil (ZFO) is an EO derived from the fruit of *Z. armatum*, which has been categorized as generally recognized as safe (GRAS). Early studies revealed that ZFO could strongly inhibit the growth of kinds of Gram-negative bacteria, such as Escherichia coli and Staphylococcus aureus [14]. More recently, Li et al. [15] showed that ZFO could inhibit the growth of Aspergillus flavus during the storage of platycladi semen. Although several instances of the biological activity of ZFO have been reported, there is no information about its antifungal function against *P. capsici*. In addition, the possible mechanism of ZFO against pathogens remains unclear.

In this study, the antifungal activity against phytopathogenic oomycetes and chemical compositions of twelve EOs from Rutaceae plants were evaluated, and the mechanism of action by which ZFO exhibited antifungal activity was investigated. Our results may contribute to the development of a safe and efficient alternative agent for the control of *P. capsici*.

## 2. Results

### 2.1. Chemical Composition of Twelve Plant EOs

**Figure 1:**
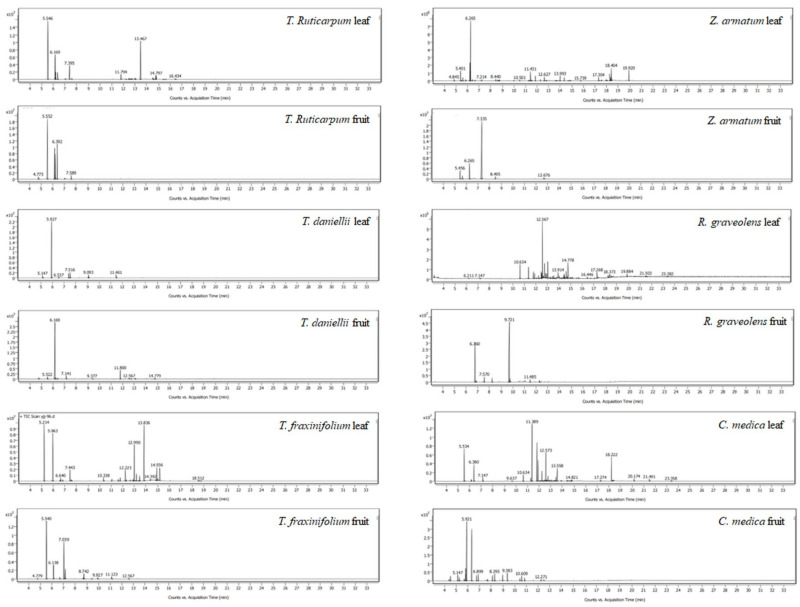
Gas chromatograms of twelve essential oils (EOs) from Rutaceae plants: *T. ruticarpum* leaf EO, *T. ruticarpum* fruit EO, *T. daniellii* leaf EO, *T. daniellii* fruit EO, *T. fraxinifolium* leaf EO, *T. fraxinifolium* fruit EO, *Z. armatum* leaf EO, *Z. armatum* fruit EO (ZFO), *R. graveolens* leaf EO, *R. graveolens* fruit EO, *C. medica* leaf EO, and *C. medica* fruit EO. See Table 1 and Tables S1–S11, and Figures S1–S12 for detailed peak characteristics.

The chemical compositions of twelve EOs from *T. ruticarpum*, *T. daniellii*, *T. fraxinifolium*, *Z. armatum*, *R. graveolens*, and *C. medica* leaves and fruits were established by GC-MS analyses. The major components of *T. ruticarpum* leaf EO were *β*-myrcene (31.03%), *E*-nerolidol (21.79%), and *β*-phellandrene (18.38%). The most abundant compound in *T. ruticarpum* fruit EO was *β*-myrcene (38.14%), followed by *β*-phellandrene (25.89%), *β*-ocimene (17.82%), and trans-*β*-ocimene (12.29%). The main component of *T. daniellii* leaf EO was *β*-myrcene, which represented 72.82% of the total EO. *D*-limonene (72.71%) was the major constituent of *T. daniellii* fruit EO. The main components of *T. fraxinifolium* leaf EO were elemol (21.64%), *α*-pinene (18.51%), *β*-myrcene (13.82%), and germacrene D (11.12%). The main constituents of *T. fraxinifolium* fruit EO were *β*-myrcene (39.83%) and 2-nonanone (27.86%). Eucalyptol (29.65%) and *D*-limonene (10.7%) were the major components of *Z. armatum* leaf EO. Linalool (73.57%) and *D*-limonene (11.31%) were the major components of ZFO. Germacrene D (17.57%) was the most abundant component in *R. graveolens* leaf EO, and the major constituents of *R. graveolens* fruit EO were 2-undecanone (62.98%) and 2-nonanone (21.6%). The main constituents of *C. medica* leaf EO were (-)-*β*-elemene (18.86%), caryophyllene (12.91%), *β*-myrcene (10.82%), and those of *C. medica* fruit EO were *D*-limonene (39.77%) and *γ*-terpinene (27.81%) (Figure 1 and Figures S1–S12). The detailed retention times, formulae, retention indices, and percentage compositions of the identified compounds are listed in Table 1 and Tables S1–S11. The extraction rates of the twelve EOs are listed in Table S12.

### 2.2. Antifungal Activities of the Twelve Plant EOs

The antifungal activities of the twelve plant EOs against phytopathogenic oomycetes were evaluated using the mycelial growth rate method. At 10 μL mL^−1^, it could easily be seen that different EOs had significantly different inhibitory effects against the tested oomycetes (Table 2). As shown in Table 2, among the twelve plant EOs, *T. daniellii* fruit EO, *T. fraxinifolium* fruit EO, and *C. medica* leaf EO exhibited the strongest antifungal activities against *P. parasitica*, with growth inhibition rates of 77.74%, 83.80%, and 80.90%, respectively. Moreover, ZFO showed the strongest inhibitory effects on all five tested oomycetes. At a concentration of 10 μL mL^−1^, ZFO completely inhibited the mycelial growth of *P. capsici*, *Py. aphanidermatum*, and *Py. ultimum*, while the inhibition rates of ZFO against *P. sojae* and *P. parasitica* were 92.83% and 95.75%, respectively (Table 2). Therefore, ZFO can be used as a potential natural antifungal substance to control diseases caused by oomycetes.

**Figure 2:**
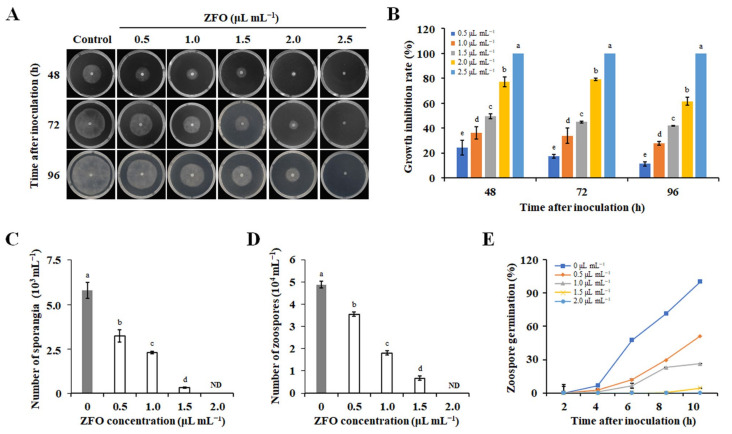
Inhibitory effect of ZFO against *Phytophthora capsici* in vitro. (**A**) Colony morphology of *P. capsici* on V8-agar plants with different concentrations of ZFO. (**B**) Statistical analysis of colony diameters. Effects of ZFO on (**C**) sporangium production, (**D**) zoospore production, and (**E**) zoospore germination of *P. capsici*. The vertical bar indicates the standard error (SE) of the mean. Different letters represent significant differences (*p* < 0.05).

To further clarify the inhibitory effect of ZFO on the mycelial growth of the pathogen, the EC_50_ values of ZFO against *P. capsici*, *Py. Aphanidermatum*, and *Py. Ultimum* were determined. Results showed that the EC_50_ value of ZFO against *P. capsici*, *Py. aphanidermatum*, and *Py. ultimum* were 0.93 μL mL^−1^, 1.12 μL mL^−1^, and 1.37 μL mL^−1^, respectively. The growth inhibition rate of ZFO against *P. capsici* followed a dose-dependent pattern (Figure 2A,B). When the concentration of ZFO was 2.5 μL mL^−1^, it completely inhibited the mycelial growth of *P. capsici*. Therefore, *P. capsici* was selected as the target organism to investigate the antifungal mechanism of ZFO.

### 2.3. ZFO Inhibited Secondary Infection of P. capsici

The effects of ZFO on zoospore production and germination were observed using optical microscopy. ZFO treatment strongly suppressed sporangia production in *P. capsici*, which was completely blocked by 2.0 μL mL^−1^ of ZFO (Figure 2C). Likewise, zoospore production of *P. capsici* was also inhibited by treatment with ZFO. The number of zoospores decreased by 86.04% when treated with 1.5 μL mL^−1^ of ZFO and was completely inhibited by 2.0 μL mL^−1^ of ZFO (Figure 2D). Zoospore germination was also sensitive to ZFO treatment. After treating with ZFO for 10 h, the zoospore germination of *P. capsici* reached 100% in the control group, and was reduced by 48.54%, 74.76%, 95.87%, and 100% with 0.5, 1.0, 1.5, and 2.0 μL mL^−1^ of ZFO, respectively (Figure 2E). These results suggested that ZFO could inhibit zoospore production as well as germination of *P. capsici*, with complete inhibition at 2.0 μL mL^−1^.

### 2.4. ZFO Inhibited P. capsici Infection in Pepper LEAVES and fruits

**Figure 3:**
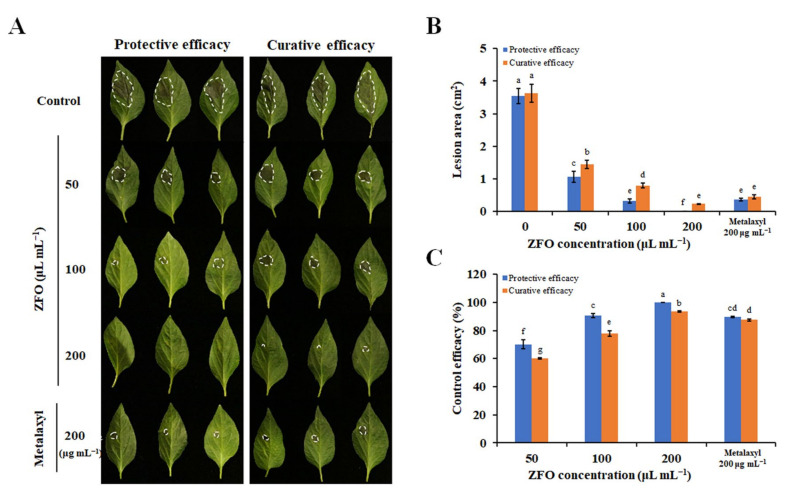
Control efficacy of ZFO against *P. capsici* in pepper leaves. The protective and curative efficacies of ZFO in controlling disease caused by *P. capsici* in pepper leaves were assessed. (**A**) Disease symptoms were photographed, and (**B**) the lesion area and (**C**) control efficacy evaluated. The vertical bar indicates the SE of the mean. Different letters represent significant differences (*p* < 0.05).

The protective and curative efficacies of ZFO in controlling disease caused by *P. capsici* in pepper leaves were assessed. The control efficacy of ZFO against *P. capsici* gradually increased with an increasing concentration of ZFO (Figure 3A). When the concentration of ZFO was 100 μL mL^−1^, the protective and curative efficacies of ZFO were respectively 90.78% and 77.94%, and when the concentration of ZFO was 200 μL mL^−1^, the protective and curative efficacies of ZFO were 100% and 93.56%, significantly higher than metalaxyl at 200 μg mL^−1^ (Figure 3B,C). It was noted that the protective efficacy of ZFO against *P. capsici* was always higher than curative efficacy.

**Figure 4:**
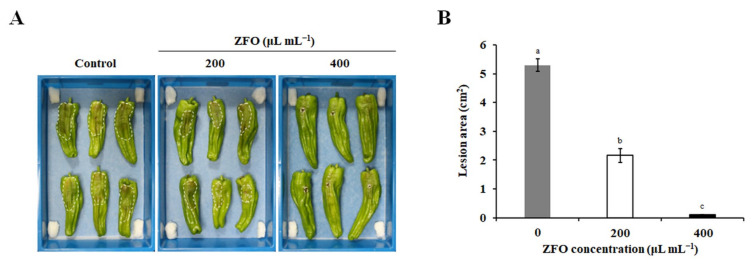
Control efficacy of the vapor phase of ZFO against *P. capsici* in pepper fruits. The protective efficacy of the vapor phase of ZFO in controlling disease caused by *P. capsici* in pepper fruits was assessed. (**A**) Disease symptoms were photographed and (**B**) the lesion areas were assessed. The vertical bar indicates the SE of the mean. Different letters represent significant differences (*p* < 0.05).

The control efficacy exerted by the vapor phase of ZFO in pepper fruits was measured. As shown in Figure 4A, ZFO vapor could effectively reduce disease caused by *P. capsici* in pepper fruits, in a dose-dependent manner. Four days after inoculation, typical dark-brown lesions with white sporangiophores caused by *P. capsici* were obvious on the control fruits. In contrast, the lesion areas in the groups treated with 200 or 400 μL mL^−1^ of ZFO were respectively reduced by 59.20% and 98.02% compared with the control group (Figure 4B). Taken together, our results demonstrate that ZFO could be an efficient alternative approach for the control of diseases caused by *P. capsici*.

### 2.5. ZFO Destroyed the Ultrastructure of P. capsici

**Figure 5:**
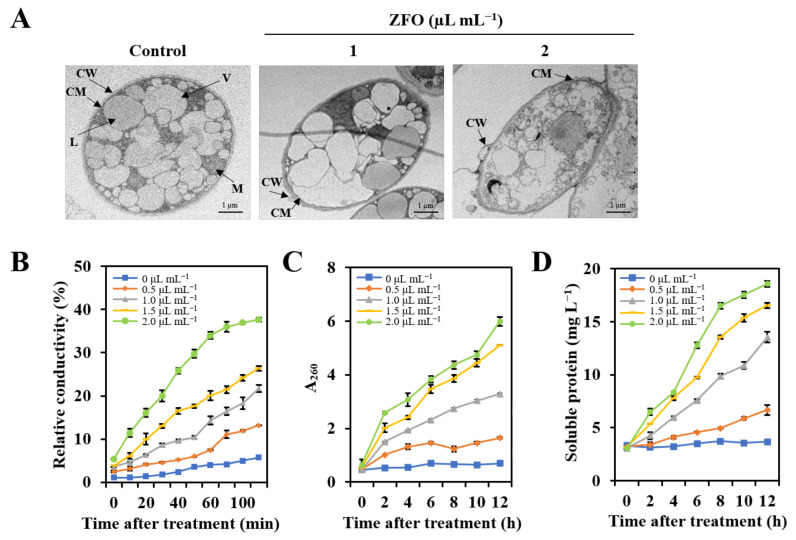
Effects of ZFO on membrane damage and intracellular leakage in *P. capsici.* (**A**) Transmission electron microscopy observations were examined to explore the effects of ZFO on the ultrastructure. CM: cell membrane; CW: cell wall; M: mitochondria; V: vacuole; L: lipidosome. (**B**) The electrical conductivity of *P. capsici* mycelia suspensions was tested to evaluate the effect of ZFO on cell membrane permeability. (**C**,**D**) The effect of ZFO on nucleic acid and protein leakage from *P. capsici* was investigated. The vertical bar indicates the SE of the mean.

TEM observations of *P. capsici* treated with ZFO revealed its effect on the ultrastructure of the mycelia. In the control group, the ultrastructure of *P. capsici* showed a clear nucleus, intact cytoplasmic organelles, a typical cytoplasmic membrane, and a compact cell wall (Figure 5A and Figure S13). However, ZFO treatment caused many dramatic ultrastructural changes. After treatment with 1 μL mL^−1^ of ZFO, the plasma membrane thickened, vacuoles increased, and the cytoplasmic matrix began to degrade. When the concentration of ZFO increased to 2 μL mL^−1^, most of the intact cytoplasmic organelles such as liposomes and mitochondria disappeared completely, and the cells gradually died (Figure 5A).

### 2.6. ZFO Damaged the Plasma Membrane of P. capsici

To investigate the inhibition mechanism of ZFO, the effect of ZFO on plasma membrane permeability in *P. capsici* was established by measuring the electrical conductivity of mycelial suspensions. As shown in Figure 5B, the conductivity values increased gradually with increasing treatment times and concentrations of ZFO. Notably, the conductivity values of the mycelia suspensions treated with 2 μL mL^−1^ of ZFO increased rapidly, 6.54-fold compared to the control after incubation for 120 min.

**Figure 6:**
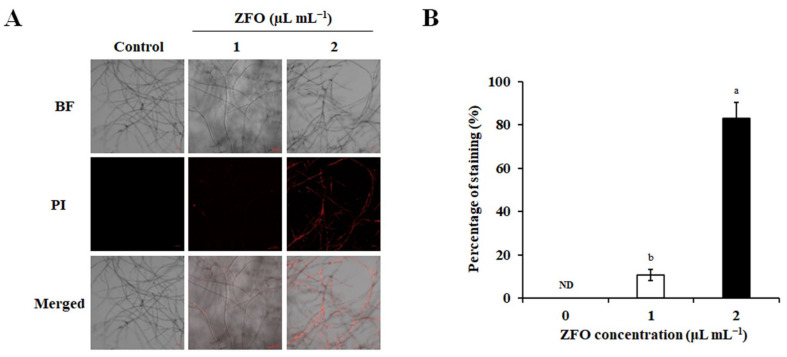
Effects of ZFO on membrane integrity of *P. capsici.* The membrane integrity of *P. capsici* was determined by PI straining. (**A**) The stained mycelia were photographed under a confocal microscope, and (**B**) percentages of stained mycelia were assessed. The vertical bar indicates the SE of the mean. Different letters represent significant differences (*p* < 0.05).

PI (Propidium Iodide) is often used to distinguish living and dead cells, because it can penetrate the plasma membrane of dead cells [16]. To further explore the effect of ZFO on the plasma membrane, we determined the membrane integrity using PI straining. As shown in Figure 6, there was no detectable fluorescence in the untreated group, while many *P. capsici* mycelia were stained after treatment with ZFO. When the concentration of ZFO was 1 μL mL^−1^, 10.81% of the mycelia lost their membrane integrity, and when the concentration increased to 2 μL mL^−1^, 82.83% of the mycelia lost their membrane integrity. These results suggest that ZFO damaged the integrity of the plasma membrane.

### 2.7. ZFO Induced Cellular Leakage of P. capsici

Damage to the plasma membrane usually leads to cell leakage. The effect of ZFO on nucleic acid and protein leakage from *P. capsici* was investigated, and the results are shown in Figure 5C,D. Leakage of nucleic acids was observed after treatment with ZFO. After incubation for 12 h at ZFO concentrations of 0.5, 1.0, 1.5, and 2.0 μL mL^−1^, the rates of nucleic acid leakage from *P. capsici* were respectively 2.37, 4.75, 7.39, and 8.68-fold compared to the control. Similarly, protein leakage also increased after exposure to ZFO. These results once again confirmed that ZFO impaired the membrane integrity of *P. capsici*.

### 2.8. ZFO Increased the Defense-Related Enzyme Activities in Pepper Fruits

**Figure 7:**
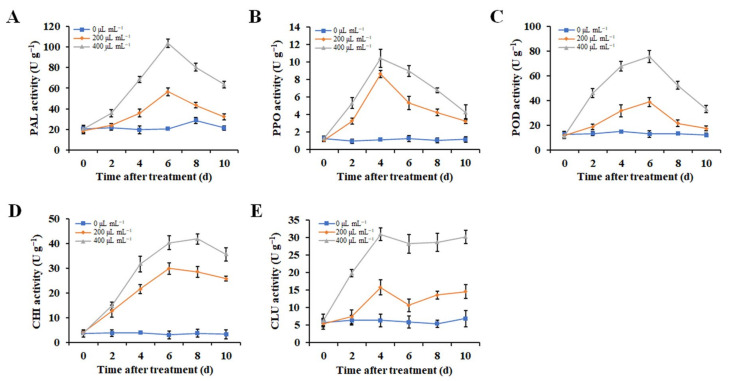
Effects of ZFO on the activities of (**A**) PAL, (**B**) PPO, (**C**) POD, (**D**) CHI, and (**E**) CLU of pepper fruits during storage. The vertical bar indicates the SE of the mean.

Next, we measured the activities of five defense-related enzymes to evaluate their possible contributions to the effect of ZFO in controlling phytophthora disease in pepper fruits. As shown in Figure 7, the activities of tested defense-related enzymes changed slightly in the control group that received no treatment, while ZFO treatments caused their enhancement. The PAL activity increased rapidly in ZFO-treated peppers and reached a peak after 6 days of storage, 200 and 400 μL mL^−1^ of TFO treatments resulted in PAL activity levels 5.15 and 2.81-fold compared with the control, respectively. After 6 days, PAL activity reduced until the end of storage. PPO and POD activities also exhibited increasing and decreasing trends during storage, reaching the highest values of 10.43 and 75.33 U g^−1^ after being treated with ZFO for 4 and 6 days, respectively. The CHI activity increased and reached a peak after 6 to 8 days of storage, 8.29 and 11.93-fold compared to the control under 200 and 400 μL mL^−1^ of TFO, respectively. CLU activity increased rapidly from 0 to 4 days, decreased slightly from 4 to 6 days, then continued to increase until the end of storage. At 10 days of storage, the respective CLU activities of TFO-treated peppers were 2.35 and 4.86-fold compared to the control under 200 and 400 μL mL^−1^ of TFO. Overall, our results indicate that ZFO increased the activities of these defense-related enzymes, suggesting an indirect strategy to reduce phytophthora disease caused by *P. capsici* in pepper fruits.

### 2.9. The Main Bioactive Components of ZFO

**Figure 8:**
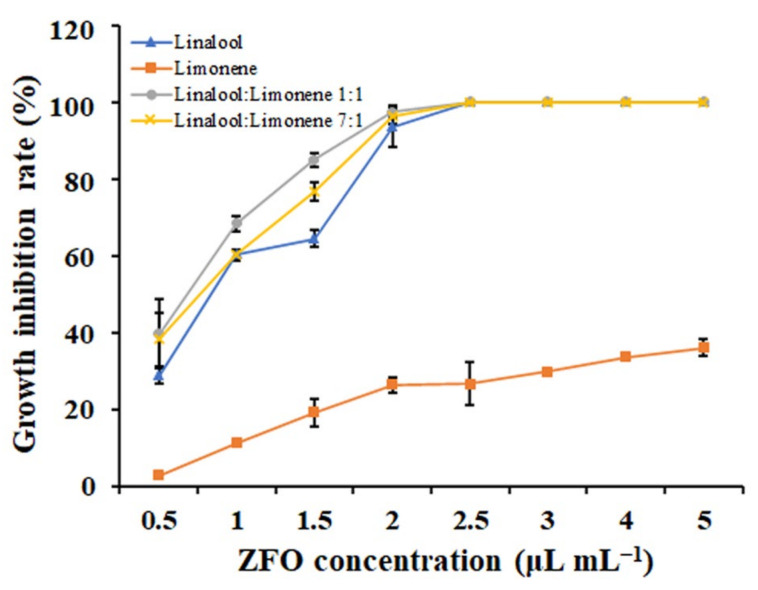
The synergistic effect of linalool and *D*-limonene against *P. capsici.* The vertical bar indicates the SE of the mean.

The inhibitory activities of EOs are due to their bioactive components [17]. Therefore, the antifungal activities of the two primary constituents identified in ZFO, linalool (73.57%) and *D*-limonene (11.31%), were evaluated. Results showed that both linalool and *D*-limonene could inhibit the mycelial growth of *P. capsici* (Figure 8). The EC_50_ values of linalool and *D*-limonene against *P. capsici* were 1.15 μL mL^−1^ and 2.94 μL mL^−1^, respectively. The synergistic effect of these two ZFO constituents against *P. capsici* was determined; mixtures were prepared at the natural ratio (7:1) or with equal amounts (1:1) of linalool and *D*-limonene. Results demonstrated that both the natural ratio and the equal mixtures had similar antifungal activities as the monomer compound linalool (Figure 8). These results indicated no synergistic effect between linalool and *D*-limonene.

## 3. Discussion

Plant EOs are naturally occurring secondary metabolites, and exhibit various biological activities [18,19]. They have many advantages including wide availability, safety, and environmental friendliness. Plants belonging to the family Rutaceae have great economic value because of their edible fruits and EOs [11]. It has previously been found that EOs extracted from Rutaceae plants possess anti-bacterial, anti-inflammatory, anti-cancer, and insecticidal properties [20,21,22]. However, the ability of EOs to inhibit oomycete pathogens has not been fully studied. In our study, we first explored by GC-MS analysis the chemical composition of twelve EOs extracted from Rutaceae plants. Chemical analysis results showed that the major components of the twelve EOs partially corroborated with previous studies of their chemistry [23,24,25,26]. However, significant differences were found in the proportions of the major constituents. It is possible that differences in the harvest season, geography, plant populations, and soil limitations were responsible for these differences [27,28].

The antifungal activity of these EOs against phytopathogenic oomycetes was then evaluated, including *P. sojae*, *P. capsici*, *P. parasitica*, *Py. aphanidermatum*, and *Py. ultimum*. Our results showed that ZFO, *T. daniellii* fruit EO, *T. fraxinifolium* fruit EO, and *C. medica* leaf EO each exhibited significant antifungal activities. Furthermore, ZFO showed the strongest inhibitory effects on all five tested oomycetes. In addition, ZFO strongly suppressed the zoospore production and germination of *P. capsici*. To our knowledge, the present study is the first report of the usage of ZFO to inhibit pathogenic oomycetes in plants. It is very important that fungicides function in vivo and in vitro. We therefore performed in vivo inoculation experiments to evaluate ZFO’s control efficacy in peppers. Our results revealed that ZFO could significantly reduce phytophthora blight disease caused by *P. capsici* in pepper leaves. In addition, the vapor of ZFO exhibited a strong protective effect against this pathogen in pepper fruits. In particular, the complete inhibitory concentration of ZFO in vivo was much higher than in vitro. This phenomenon might be due to interactions between ZFO and the host plants. Similar results were reported in studies of pterostilbene and esculetin [29,30]. Moreover, it is also worth noting that ZFO, due to its volatility, could be applied by fumigation to achieve antifungal activities. This advantage expands its range of use.

To explore the mechanism of action by which ZFO inhibited the growth of *P. capsici*, we determined the integrity of the plasma membrane in this pathogen after exposure to ZFO. TEM observation results revealed that after treatment with ZFO, the plasma membrane was destroyed, intact cytoplasmic organelles disappeared, and the cells gradually died. The results suggest that the plasma membrane of *P. capsici* might be the target of ZFO. We also examined the effects of ZFO on cell membrane permeability and leakage of the intracellular contents of *P. capsici*. Our results demonstrated that the electrical conductivity strongly increased and significant leakage of the intracellular contents of *P. capsici* was observed after treatment with ZFO. Many plant EOs includng tea tree oil and *Perilla frutescens* EO have been reported to damage the cell membrane, affect the morphology and ultrastructure, destroying cell-membrane integrity, and inhibiting the mycelial growth of pathogens [31,32]. The results of the present study are strongly supported by the findings cited above, and we attribute the antifungal effect of ZFO against *P. capsici* to the damage caused to the plasma membrane.

When faced with biological stress, plants enhance their resistance to disease by increasing the activities of defense-related enzymes to protect themselves from the invasion of phytopathogens. PAL is a key enzyme in the phenylpropanoid metabolic pathway, which regulates the biosynthesis of phenylpropanoid compounds such as lignin, phenolics, and flavonoids, and plays an important role in plants’ disease resistance [33]. PPO is an enzyme that participates in the secondary metabolism of plants, which can convert phenolics into more toxic quinones and increase toxicity tolerance [34]. POD plays a major role in cell-wall biosynthesis and enhances disease resistance by increasing the lignin content in the cell wall. Meanwhile, POD can also help to remove ROS by decomposing H_2_O_2_ into H_2_O [35,36]. CHI and GLU have been reported to degrade chitin and *β*-1,3-glucan in the pathogenic fungal cell wall and stimulate the disease resistance of host plants [37]. In the present study, we demonstrated that the activities of PAL, PPO, POD, CHI, and CLU in pepper fruits were significantly increased after treatment with ZFO. Consistent with our findings, previous studies also demonstrated that some plant EOs or their volatile compounds could enhance disease resistance of host plants through increasing activities of defense-related enzymes [38,39]. Therefore, these findings suggest together that ZFO could activate the defensive system of pepper, which may be responsible for controlling phytophthora disease caused by *P. capsici*.

The inhibitory activities of EOs are determined by their bioactive components. Next, we tested the inhibitory effect of the two main constituents of ZFO, linalool and *D*-limonene. Our results showed that both linalool and *D*-limonene could significantly inhibit the mycelial growth of *P. capsici*. We suggest that the antifungal activity of ZFO against *P. capsici* might be due to the content of linalool and *D*-limonene. In addition to ZFO, *T. daniellii* fruit EO, *T. fraxinifolium* fruit EO, and *C. medica* leaf EO also exhibited good antifungal activities. Chemical composition results showed that the main constituent of *T. daniellii* fruit EO was *D*-limonene (72.71%), and *T. fraxinifolium* leaf EO contained *D*-limonene (9.58%) and linalool (7.95%). These results strongly indicate that linalool and *D*-limonene were the primary effective components. Linalool has been reportedly used as an antimicrobial agent due to its strong antimicrobial activities [40]. Previous studies have shown the antifungal activity of linalool against various phytopathogenic fungi and bacteria, such as *Rhizoctonia solani*, *Fusarium oxysporum*, *Penicillium digitatum*, and *Colletotrichum lindemuthianum* [41,42]. Studies on limonene have also revealed its good antifungal effect against phytopathogens, including *Listeria monocytogenes* and *Candida tropicalis* [43,44]. Further research is needed to explore the possible antifungal mechanisms of linalool and *D*-limonene against *P. capsici.*

## 4. Materials and Methods

### 4.1. Oomycete Pathogens and Chemicals

The oomycete pathogens including *P. capsici*, *P. sojae*, *P. parasitica*, *Py. aphanidermatum*, and *Py. ultimum* used in this study were kindly provided by Prof. Daolong Dou at Nanjing Agricultural University, China. All strains were cultivated on V8-agar plates, which contained 10% V8 juice, 0.02% CaCO_3_, and 1.5% agar at 25 °C before use.

All chemicals and anhydrous solvents were purchased from commercial suppliers. Metalaxyl (98%), linalool (98%), and *D*-limonene (95%) were obtained from Aladdin Biotechnology (Shanghai, China). Other chemicals and reagents of analytical grade were obtained from Solarbio Science and Technology (Beijing, China).

### 4.2. Plant Materials

Leaves and fruits of *T. ruticarpum* were collected from Tongren, Guizhou Province (27°72′ N, 109°18′ E) in August 2018. Leaves and fruits of *T. daniellii* and *Z. armatum* were collected from Chengdu, Sichuan Province (30°67′ N, 104°06′ E) in August 2019. Leaves and fruits of *T. fraxinifolium* were collected from Dali, Yunnan Province (25°28′ N, 99°59′ E) in August 2019. Leaves and fruits of *R. graveolens* and *C. medica* were collected from Kunming, Yunnan Province (24°25′ N, 102°15′ E) in July and August 2019. Plant materials were identified by Prof. Changqi Yuan (Institute of Botany, Jiangsu Province and Chinese Academy of Sciences). The collected plant materials were subsequently used for EO extraction.

Pepper (cv. sujiao 5) leaves were obtained from an experimental field at the Institute of Botany, Jiangsu Province, and Chinese Academy of Sciences, China. After surface sterilization with 2% NaClO, pepper leaves of the same size and age were selected for testing. Pepper fruits at commercial maturity were also harvested from the experimental field. All fruits were free of infection or injury, and of the same size. Fruits were surface sterilized before use in inoculation experiments.

### 4.3. EO Extraction

The extraction of EOs from *T. ruticarpum*, *Z. armatum*, *T. daniellii*, *T. fraxinifolium*, *R. graveolens*, and *C. medica* was implemented as described by Zhao et al. [18]. Briefly, the plant materials were extracted using a hydrodistillation method with a Clevenger-type apparatus. After distillation for 5 h, the resulting EOs were dried over anhydrous Na_2_SO_4_. The extracted EOs were then collected in glass vials and kept at 4 °C before use. Solution containing 0.05% Tween-80 was used in the subsequent tests to help dissolve EOs in V8 culture medium or water.

### 4.4. EO Identification

Gas chromatography–mass spectrometry (GC-MS) was conducted to analyze the volatile constituents of the EOs (Agilent 6890/5973, Agilent Technologies, Santa Clara, CA, USA), with a chromatographic column (HP-5MS capillary column, 30 m × 0.25 mm × 0.25 μm). Helium was used as the carrier gas at a constant flow rate of 1.0 mL min^−1^. At first, the column furnace was kept at 40 °C for 3 min, increasing to 120 °C at a rate of 5 °C min^−1^, and maintained for 3 min. The temperature was then increased to 280 °C at a rate of 30 °C min^−1^, and maintained for 5 min. For the MS analysis, the injector and detector temperatures were both 250 °C, the ion source temperature was 230 °C, the mass scan range was 33–800 amu, and the ionization energy was 70 eV. The retention indices were determined in relation to a homologous series of n-alkanes (C_8_–C_24_) under the same conditions. Identification of volatile compounds was conducted by comparison of the retention index values with those reported in the literature, and of the mass spectra with those in the National Institute of Standards and Technology (NIST) spectral library. Mass spectrum data were determined of compounds identified by NIST17.L with similarity of more than 70%.

### 4.5. Effects of EOs on Mycelial Growth

The mycelial growth rate method was employed to evaluate the growth inhibition rate of the EOs [18]. The EOs were diluted to 10 μL mL^−1^ in V8-agar, which contained 0.05% Tween-80. Mycelial plugs of oomycetes obtained from the edges of 2- to 5-day-old colonies were inoculated onto the center of the plates. For the investigation of the median effective concentration (EC_50_) of ZFO against *P. capsici*, *Py. aphanidermatum*, and *Py. ultimum,* samples were diluted to 0.5, 1.0, 1.5, 2.0, or 2.5 μL mL^−1^ in V8-agar (containing 0.05% Tween-80). V8-agar amended with the same amount of Tween-80 was used as the control. The plates were cultured at 25 °C for 2 to 5 days, the mycelial diameters were determined, and the growth inhibition rates were estimated. Each group consisted of three plates and the experiments were repeated three times in their entirety.

### 4.6. Effects of ZFO on Secondary Infection of P. capsici

Next, we tested the effects of the ZFO on zoospore production as well as germination of *P. capsici*, as described previously [30]. Briefly, mycelial plugs of *P. capsici* were detached and placed in 10 mL of V8 medium, and the cultures were incubated at 25 °C for 2 days. The mycelia were washed and resuspended in 10 mL of sterilized tap water, which contained 0, 0.5, 1.0, 1.5, or 2.0 μL mL^−1^ of ZFO. After incubation at 25 °C for 24 h, sporangia were counted under a microscope (Olympus IX-71, Tokyo, Japan). For the determination of zoospore production, equivalent quantities of sporangia were transferred into 10 mL of sterilized tap water, which contained 0, 0.5, 1.0, 1.5, or 2.0 μL mL^−1^ of ZFO. Then, the cultures were placed at 4 °C for 1 h, and transferred to 25 °C. For the determination of zoospore germination, equal quantities of zoospores were cultured in 10 mL of V8 liquid medium amended with 0, 0.5, 1.0, 1.5, or 2.0 μL mL^−1^ of ZFO. The germination rates of the zoospores were measured at 2, 4, 6, 8, and 10 h using the microscope. The entire experiment was repeated four times.

### 4.7. In Vivo Antifungal Bioassay of ZFO

Protective and curative activities of ZFO against *P. capsici* were tested in pepper as previously described [45]. After surface sterilization, pepper leaves of the same size and age were selected. ZFO solutions of different concentrations (50, 100, or 200 μL mL^−1^ in sterile distilled water) were prepared. Sterile distilled water with 200 μg mL^−1^ of metalaxyl as reference fungicide was used as the control. *P. capsici* zoospore suspensions (1 × 10^5^ zoospores mL^−1^) were prepared as described in Section 2.5. To determine protective activity, ZFO or metalaxyl dilutions was sprayed on the pepper leaves, then after 24 h the surface of each pepper leaf was inoculated with 10 μL of the zoospore solutions. For assessment of curative activity, the pepper leaves were sprayed with the treatments after inoculation with zoospore solutions of *P. capsici* for 24 h. These leaves were cultured under a long-day (16:8) photoperiod at 25 °C for 3 days. Each group consisted of at least 20 leaves and the experiment was performed three times.

EOs can evaporate from surfaces because of their volatile nature [46]. Therefore, the control efficacy of the vapor phase of ZFO against *P. capsici* in pepper fruits was tested as described by Hosseini et al. [47] with minor modification. Briefly, the ZFO was diluted to 200 or 400 μL mL^−1^ in sterile distilled water. Then, four cotton balls absorbed 1 mL of ZFO dilutions and were placed at the edge of a transparent plastic container. Pepper fruits were wounded and 10 μL of the zoospore solutions were inoculated on the surface. Six fruits were placed in one container. To prevent vapor leakage, all the plastic containers were sealed with parafilm. After being cultured at 25 °C for 4 days, the lesion areas were measured and analyzed. Control efficacy = (lesion area of water treatment − lesion area of drug treatment)/lesion area of water treatment. These experiments were repeated four times.

### 4.8. Transmission Electron Microscopy (TEM) Analysis

TEM observations were performed to evaluate the effects of ZFO on the ultrastructure of *P. capsica*, as described previously [9]. Mycelial plugs of *P. capsici* were inoculated on V8-agar plates that contained 0, 1.0, or 2.0 μL mL^−1^ of ZFO. The plates were cultured at 25 °C for 4 days, and the mycelial tips from fresh cultures were collected. The samples were totally fixed in 2.5% glutaraldehyde and then in 0.1 M osmium tetroxide. The samples were subsequently dehydrated in an ethanol series for 20 min each. After embedding in Epon resin, the samples cut into ultrathin sections and stained with uranyl acetate and lead citrate. The ultrastructure of *P. capsici* was observed using a Talos-F200C transmission electron microscope (Thermo Fisher Scientific, Waltham, MA, USA). The experiment was conducted three times.

### 4.9. Effects of ZFO on Membrane Damage of P. capsici

The effects of ZFO on the extracellular electrical conductivity were evaluated following the method described by Wang et al. [30]. Briefly, mycelial plugs of *P. capsici* were cultured in 100 mL of V8 liquid medium and shaken at 25 °C for 4 days. Then, the mycelia were harvested with sterile gauze and resuspended in sterile distilled water, which contained 0, 0.5, 1.0, 1.5, or 2.0 μL mL^−1^ of ZFO. After being cultured and shaken at 25 °C for up to 100 min, the extracellular electrical conductivities of *P. capsici* were measured at 0, 10, 20, 30, 40, 50, 60, 80, 100, and 120 min. The samples were boiled after 120 min and the final extracellular electrical conductivity was recorded. Relative electrical conductivity = electrical conductivity/final electrical conductivity.

The fluorescent dye PI is commonly used for determining cell viability. The PI staining experiment was carried out using the method reported by He et al. [16]. Mycelial plugs of *P. capsici* were detached and put into 10 mL of V8 liquid medium, then the cultures were incubated at 25 °C for 2 days. ZFO at final concentrations of 0, 1.0, or 2.0 μL mL^−1^ was then added into the V8 liquid medium. After incubation for a further day at 25 °C, the mycelia were washed and stained with 200 μM PI. The stained mycelia were observed by using a confocal microscope (LSM880, Carl Zeiss, Jena, Germany). Each experiment was performed four times.

### 4.10. Effects of ZFO on Intracellular Leakage of P. capsici

The intracellular leakage of the pathogen was determined as reported by Wang et al. [38]. Mycelial plugs of *P. capsici* were cultured in 100 mL of V8 liquid medium and shaken at 25 °C for 4 days. Then, the mycelia were washed and resuspended in sterile distilled water, which contained 0, 0.5, 1.0, 1.5, or 2.0 μL mL^−1^ of ZFO. After being cultured with shaking at 25 °C for up to 12 h, the supernatants were collected at 0, 2, 4, 6, 8, 10, and 12 h by filtering through sterile gauze, and the DNA and protein content were determined. The release of DNA and protein was quantified by determining the absorbance at 260 nm and with Coomassie brilliant blue staining, respectively. These experiments were performed in triplicate.

### 4.11. Effects of ZFO on Defense-Related Enzyme Activities in Pepper Fruits

Pepper fruits were fumigated with ZFO at concentrations of 0, 200, or 400 μL mL^−1^ as described in Section 4.6. After fumigation for up to 10 days, the pepper fruits were collected at 0, 2, 4, 6, 8, and 10 days, and the activity levels of phenylalanine ammonia lyase (PAL), polyphenol oxidase (PPO), peroxidase (POD), chitinase (CHI), and *β*-1,3-glucanase (GLU) in the pepper fruits were measured according to methods reported previously [38,39]. Briefly, flesh tissue (2.0 g) was homogenized with 3.0 mL of 0.1 M acetate buffer (pH 5.5) and then centrifuged at 12,000× *g* for 30 min. The supernatant was used as the crude enzyme extract. The activities of PAL, PPO, POD, CHI, and GLU were determined using appropriate kits purchased from Nanjing Jiancheng Bioengineering Institute (Nanjing, China). The experiment was conducted four times.

### 4.12. Statistical Analysis

There were more than three replicates for each treatment and all experiments were implemented at least in triplicate. We performed one-way analysis of variance (ANOVA) to analyze the data using the SPSS software (v14.0 SPSS Inc., Chicago, IL, USA), and Fisher’s least significant difference (LSD) test was applied to reveal the differences between experimental groups. Differences at *p* < 0.05 represented significant differences.

## 5. Conclusions

In this study, we investigated the chemical composition of twelve EOs from Rutaceae plants, and their antifungal activity against phytopathogenic oomycetes. The results demonstrated the inhibitory activity of ZFO against *P. capsici* in vitro and in planta. The antifungal mechanism of ZFO was attributed to destruction of the ultrastructure, damage to membrane permeability, and leakage of the cytoplasmic contents. In addition, ZFO could enhance the activities of defense-related enzymes to suppress development of phytophthora disease in pepper fruits. Two major components of ZFO (linalool and *D*-limonene) were shown to be the primary effective agents. As far as we know, this is the first work to reveal the inhibitory effect of ZFO against phytopathogenic oomycetes and report its efficacy for reducing disease severity. Our study provides evidence that ZFO is a potential candidate agent for management of disease caused by *P. capsici*.

## Figures and Tables

**Table 1:** Chemical composition of *Z. armatum* fruit essential oil as shown by GC-MS analysis.

Peak Number	Retention Time (min)	Formula	Compound	Molecular Weight	Retention Indices ^a^	Retention Indices ^b^	Areas ^c^ (%)
1	4.78	C_10_H_16_	2-thujene	136	968	978	0.1
2	4.9	C_10_H_16_	(-)-*α*-pinene	136	942	936	0.25
3	5.3	C_8_H_16_O	2,4-dimethyl-cyclohexanol	128	1032	1032	0.12
4	5.46	C_10_H_16_	sabinene	136	967	946	5.79
5	5.53	C_10_H_16_	(-)-*β*-pinene	136	943		0.27
6	5.65	C_10_H_16_	*β*-myrcene	136	983	984	2.04
7	5.82	C_8_H_16_O	octanal	128	982	1004	0.06
8	6.08	C_10_H_16_	*α*-terpinene	136	1010	1010	0.33
9	6.27	C_10_H_16_	*D*-limonene	136	1018		11.31
10	6.5	C_10_H_16_	*β*-ocimene	136	1037	1259	0.41
11	6.7	C_10_H_16_	*γ*-terpinene	136	1050	1240	0.5
12	6.84	C_10_H_18_O	4-thujanol	154	1093	1109	0.16
13	7.15	C_10_H_16_	*α*-terpinolene	136	1079	1089	0.23
14	7.33	C_10_H_18_O	linalool	154	1086	1103	73.57
15	7.47	C_10_H_18_O	isothujol	154	1152	1170	0.1
16	7.61	C_10_H_16_O	thujone	152	1089	1105	0.07
17	7.67	C_10_H_18_O	*trans*-*p*-2-menthen-1-ol	154	1112	1108	0.09
18	7.92	C_10_H_18_O	*cis*-*p*-menth-2-en-1-ol	154	1111	1126	0.07
19	8.07	C_10_H_18_O	citronellal	154	1134	1153	0.08
20	8.29	C_10_H_20_O	levomenthol	156	1160	1172	0.12
21	8.49	C_10_H_18_O	terpinen-4-ol	154	1164	1167	1.39
22	8.68	C_10_H_18_O	*α*-terpineol	154	1175	1183	0.37
23	9.51	C_11_H_18_O_2_	linalyl formate	182	1206	1215	0.08
24	9.61	C_10_H_16_O	piperiton	152	1233	1255	0.09
25	11.91	C_15_H_24_	caryophyllene	204	1419	1423	0.26
26	12.34	C_15_H_24_	humulene	204	1451	1436	0.07
27	12.68	C_15_H_24_	germacrene D	204	1477	1482	0.92
28	12.87	C_15_H_24_	bicylogermacrene	204	1492	1057	0.25
29	14.89	C_15_H_26_O	*α*-cadinol	222	1642	1635	0.06
Total							99.16

Retention indices ^a^ = calculated retention index; Retention indices ^b^ = retention index reported previously; ^c^ The relative proportions of the essential oil constituents.

**Table 2:** Effects of twelve essential oils on the mycelial growth of five oomycetes at 10 μL mL^−1^.

Species	Organs	Growth Inhibition Rate (%)				
		*P. capsici*	*P. sojae*	*P. parasitica*	*Py. aphanidermatum*	*Py. ultimum*
*T. ruticarpum*	Leaf	39.02 ± 5.41	9.40 ± 2.55	20.90 ± 5.72	0	60.90 ± 5.02
	Fruit	14.63 ± 2.17	5.20 ± 2.91	25.00 ± 3.28	0	43.50 ± 5.44
*T. daniellii*	Leaf	27.91 ± 4.62	34.67 ± 4.64	0	22.00 ± 3.27	11.90 ± 2.76
	Fruit	61.67 ± 1.06	35.52 ± 2.12	77.74 ± 2.03	0	22.17 ± 4.21
*T. fraxinifolium*	Leaf	25.00 ± 2.58	31.50 ± 3.68	47.10 ± 5.70	5.00 ± 3.25	9.78 ± 2.83
	Fruit	55.10 ± 4.71	25.80 ± 2.87	83.80 ± 6.45	15.00 ± 2.76	29.35 ± 3.16
*Z. armatum*	Leaf	27.89 ± 3.84	51.68 ± 4.21	33.80 ± 3.98	25.85 ± 3.66	43.38 ± 3.75
	Fruit	100	92.83 ± 5.24	95.75 ± 5.32	100	100
*R. graveolens*	Leaf	0	0	0	12.37 ± 3.23	11.62 ± 2.12
	Fruit	17.52 ± 3.71	10.32 ± 1.37	27.37 ± 1.94	3.71 ± 0.77	42.17 ± 4.60
*C. medica*	Leaf	39.02 ± 4.38	9.40 ± 2.10	80.90 ± 4.35	0	60.90 ± 5.77
	Fruit	42.19 ± 3.63	23.71 ± 2.76	54.31 ± 2.96	9.84 ± 0.31	39.25 ± 1.03

## Supplementary Materials

The supporting information can be downloaded at: https://www.mdpi.com/article/10.3390/molecules27238636/s1. Table S1: Chemical composition of *Tetradium ruticarpum* leaf essential oil as shown by GC-MS analysis; Table S2: Chemical composition of *Tetradium ruticarpum* fruit essential oil as shown by GC-MS analysis; Table S3: Chemical composition of *Tetradium daniellii* leaf essential oil as shown by GC-MS analysis; Table S4: Chemical composition of *Tetradium daniellii* fruit essential oil as shown by GC-MS analysis; Table S5: Chemical composition of *Tetradium fraxinifolium* leaf essential oil as shown by GC-MS analysis; Table S6: Chemical composition of *Tetradium fraxinifolium* fruit essential oil as shown by GC-MS analysis; Table S7: Chemical composition of *Zanthoxylum armatum* leaf essential oil as shown by GC-MS analysis; Table S8: Chemical composition of *Ruta graveolens* leaf essential oil as shown by GC-MS analysis; Table S9: Chemical composition of *Ruta graveolens* fruit essential oil as shown by GC-MS analysis; Table S10: Chemical composition of *Citrus medica* leaf essential oil as shown by GC-MS analysis; Table S11: Chemical composition of *Citrus medica* fruit essential oil as shown by GC-MS analysis; Table S12: Extraction yield of essential oils from twelve materials; Figure S1: GC-MS chromatogram of *Tetradium ruticarpum* leaf essential oil. The corresponding peaks were marked with the represented substances as shown in Table S1; Figure S2: GC-MS chromatogram of *Tetradium ruticarpum* fruit essential oil. The corresponding peaks were marked with the represented substances as shown in Table S2; Figure S3: GC-MS chromatogram of *Tetradium daniellii* leaf essential oil. The corresponding peaks were marked with the represented substances as shown in Table S3; Figure S4: GC-MS chromatogram of *Tetradium daniellii* fruit essential oil. The corresponding peaks were marked with the represented substances as shown in Table S4; Figure S5: GC-MS chromatogram of *Tetradium fraxinifolium* leaf essential oil. The corresponding peaks were marked with the represented substances as shown in Table S5; Figure S6: GC-MS chromatogram of *Tetradium fraxinifolium* fruit essential oil. The corresponding peaks were marked with the represented substances as shown in Table S6; Figure S7: GC-MS chromatogram of *Zanthoxylum armatum* leaf essential oil. The corresponding peaks were marked with the represented substances as shown in Table S7; Figure S8: GC-MS chromatogram of *Zanthoxylum armatum* fruit essential oil. The corresponding peaks were marked with the represented substances as shown in Table 1; Figure S9: GC-MS chromatogram of *Ruta graveolens* leaf essential oil. The corresponding peaks were marked with the represented substances as shown in Table S8; Figure S10: GC-MS chromatogram of *Ruta graveolens* fruit essential oil. The corresponding peaks were marked with the represented substances as shown in Table S9; Figure S11: GC-MS chromatogram of *Citrus medica* leaf essential oil. The corresponding peaks were marked with the represented substances as shown in Table S10; Figure S12: GC-MS chromatogram of *Citrus medica* fruit essential oil. The corresponding peaks were marked with the represented substances as shown in Table S11; Figure S13: Transmission electron microscopy observations were examined to explore the effects of ZFO on the ultrastructure. CM: cell membrane; CW: cell wall; M: mitochondria; V: vacuole; and L: lipidosome.
