# Supplementary material for: Chemical Composition and Antifungal Activity of Zanthoxylum armatum Fruit Essential Oil against Phytophthora capsici
Source: Molecules. 2022 Dec 6;27(23):8636. doi: 10.3390/molecules27238636 (PMC9740196; doi:10.3390/molecules27238636)
Supplement: Supplementary file 1 [file molecules-27-08636-s001.zip › molecules-2068950-supplementary.pdf]

Chemical Composition and Antifungal Activity of *Zanthoxylum armatum* Fruit Essential Oil against *Phytophthora capsici*

Jingjing Yang [1,2,†], Qizhi Wang [1,2,†], Linwei Li [1], Pirui Li [1], Min Yin [1], Shu Xu [1], Yu Chen [1], Xu Feng [1] and Bi Wang [1*]

[1] Jiangsu Key Laboratory for the Research and Utilization of Plant Resources, Jiangsu Province Engineering Research Center of Eco-cultivation and High-value Utilization of Chinese Medicinal Materials, Institute of Botany, Jiangsu Province and Chinese Academy of Sciences (Nanjing Botanical Garden Mem. Sun Yat-Sen), Nanjing 210014, China
[2] Nanjing University of Chinese Medicine, Nanjing 210023, China
* Correspondence: Bi Wang (wangbi@cnbg.net); Tel: +86-25-8434-7074
† These authors contributed equally to the paper.

Table S1. Chemical composition of *Tetradium ruticarpum* leaf essential oil as shown by GC-MS analysis.

Peak number	Retention time (min)	Compound	Formula	Molecular Weight	Retention indices [a]	Retention indices [b]	Areas (%)
1	5.55	β-myrcene	$C_{10}H_{16}$	136	983	998	31.03
2	5.78	α-phellandrene	$C_{10}H_{16}$	136	998	997	0.19
3	6.17	β-phellandrene	$C_{10}H_{16}$	136	1021	1174	18.38
4	6.22	trans-β-ocimene	$C_{10}H_{16}$	136	1038	1048	3.35
5	6.37	β-ocimene	$C_{10}H_{16}$	136	1037	1037	3.21
6	7.03	α- terpinolen	$C_{10}H_{16}$	136	1079	1089	0.38
7	7.15	linalool	$C_{10}H_{18}O$	154	1086	1095	0.24
8	7.4	(*E*)-4,8-dimethyl-1,3,7-nonatriene	$C_{11}H_{18}$	150	1075	1116	6.17
9	11.79	caryophyllene	$C_{15}H_{24}$	204	1419	1423	2.21
10	11.9	α-bergamotene	$C_{15}H_{24}$	204	1434	1433	0.23
11	12.1	(*E*)-β-famesene	$C_{15}H_{24}$	204	1448	1468	0.22
12	12.23	humulene	$C_{15}H_{24}$	204	1451	1457	0.33
13	12.47	γ-muurolene	$C_{15}H_{24}$	204	1472	1435	0.64
14	12.56	germacrene D	$C_{15}H_{24}$	204	1477	1428	0.35
15	12.65	β-eudesmene	$C_{15}H_{24}$	204	1482	1482	0.34
16	12.71	cubebol	$C_{15}H_{26}O$	222	1510	1515	0.29
17	12.74	δ-guaiene	$C_{15}H_{24}$	204	1502	1508	0.45
18	12.8	β-bisabolene	$C_{15}H_{24}$	204	1500	1514	0.14
19	12.85	δ-cadinene	$C_{15}H_{24}$	204	1516	1498	0.12
20	12.95	γ-cadinene	$C_{15}H_{24}$	204	1507	1513	0.31
21	13.13	(-)-spathulenol	$C_{15}H_{24}O$	220	1577	1582	0.12
22	13.47	*E*-nerolidol	$C_{15}H_{26}O$	222	1549	1571	21.79
23	13.92	(-)-globulol	$C_{15}H_{26}O$	222	1530	1580	0.27
24	14.46	1,10-diepicubenol	$C_{15}H_{26}O$	222	1609	1623	0.28
25	14.52	γ-eudesmol	$C_{15}H_{26}O$	222	1618	1617	1.09
26	14.62	T-cadinol	$C_{15}H_{26}O$	222	1627	1644	0.55
27	14.78	β-eudesmol	$C_{15}H_{26}O$	222	1636	1639	1.52
28	14.8	α-eudesmol	$C_{15}H_{26}O$	222	1643	1643	2.1
29	15.07	α-bisabolol	$C_{15}H_{26}O$	222	1668	1688	0.19
30	15.39	farnesyl alcohol	$C_{15}H_{26}O$	222	1682	2350	0.47
total							96.96

Retention indices [a]-calculated retention index, Retention indices [b]-retention index reported from previous reports.

Table S2. Chemical composition of *Tetradium ruticarpum* fruit essential oil as shown by GC-MS analysis.

Peak number	Retention time (min)	Compound	Formula	Molecular Weight	Retention indices [a]	Retention indices [b]	Areas (%)
1	4.77	α-pinene	$C_{10}H_{16}$	136	933	937	1.63
2	5.33	sabinen	$C_{10}H_{16}$	136	967	946	0.24
3	5.41	(-)-β-pinene	$C_{10}H_{16}$	136	943		0.12
4	5.55	β-myrcene	$C_{10}H_{16}$	136	983	984	38.14
5	6.17	β-phellandrene	$C_{10}H_{16}$	136	1021	1029	25.89
6	6.22	*trans*-β-ocimene	$C_{10}H_{16}$	136	1038	1048	12.29
7	6.39	β-ocimene	$C_{10}H_{16}$	136	1037	1037	17.82
8	7.03	α- terpinolen	$C_{10}H_{16}$	136	1079	1089	0.54
9	7.15	linalool	$C_{10}H_{18}O$	154	1086	1095	0.28
10	7.53	(E)-*p*-menth-2-en-1-ol	$C_{10}H_{18}O$	154	1112	1108	0.08
11	8.56	α-terpineol	$C_{10}H_{18}O$	154	1175	1183	0.08
12	10.88	nerol acetate	$C_{12}H_{20}O_2$	196	1343	1367	0.12
13	11.36	β-elemene	$C_{15}H_{24}$	204	1388	1389	0.11
14	11.79	caryophyllene	$C_{15}H_{24}$	204	1419	1423	0.24
15	12.57	germacrene D	$C_{15}H_{24}$	204	1477	1482	0.16
16	13.44	E-nerolidol	$C_{15}H_{26}O$	222	1549	1571	0.16
total							97.9

Retention indices [a]-calculated retention index, Retention indices [b]-retention index reported from previous reports.

Table S3. Chemical composition of *Tetradium daniellii* leaf essential oil as shown by GC-MS analysis.

Peak number	Retention time (min)	Compound	Formula	Molecular Weight	Retention indices [a]	Retention indices [b]	Areas (%)
1	5.15	α-pinene	$C_{10}H_{16}$	136	933	937	2.88
2	5.93	β-myrcene	$C_{10}H_{16}$	136	983	984	72.82
3	6.07	octanal	$C_8H_{16}O$	128	982	1004	0.15
4	6.19	hexyl acetate	$C_8H_{16}O_2$	144	995	996	0.18
5	6.54	β-phellandrene	$C_{10}H_{16}$	136	1021	1029	1.09
6	6.58	*trans*-β-ocimene	$C_{10}H_{16}$	136	1038	1048	0.61
7	6.74	*cis*-β-ocimene	$C_{10}H_{16}$	136	1028	1035	0.31
8	7.38	2-nonanone	$C_9H_{18}O$	142	1071	1092	4.73
9	7.52	linalool	$C_{10}H_{18}O$	154	1086	1095	5.69
10	8.92	α-terpineol	$C_{10}H_{18}O$	154	1175	1183	0.19
11	9.05	decanal	$C_{10}H_{20}O$	156	1185	1205	0.69
12	9.75	*cis*-geraniol	$C_{10}H_{18}O$	154	1213	1230	0.13
13	9.99	α-citral	$C_{10}H_{16}O$	152	1249	1270	0.3
14	10.28	2-undecanone	$C_{11}H_{22}O$	170	1273	1294	0.21
15	11.06	citronellol acetate	$C_{12}H_{22}O_2$	198	1335	1354	0.24
16	11.21	nerol acetate	$C_{12}H_{20}O_2$	196	1343	1367	0.1
17	11.46	geranyl acetate	$C_{12}H_{20}O_2$	196	1361	1396	3.71
18	11.79	n-decyl acetate	$C_{12}H_{24}O_2$	200	1393	1409	0.26
19	13.74	elemol	$C_{15}H_{26}O$	222	1537	1550	0.51
20	14.87	γ-eudesmol	$C_{15}H_{26}O$	222	1618	1617	0.11
21	17.52	m-camphorene	$C_{20}H_{32}$	272	1944	1960	0.24
total							95.15

Retention indices [a]-calculated retention index, Retention indices [b]-retention index reported from previous reports.

Table S4. Chemical composition of *Tetradium daniellii* fruit essential oil as shown by GC-MS analysis.

Peak number	Retention time (min)	Compound	Formula	Molecular Weight	Retention indices [a]	Retention indices [b]	Areas (%)
1	4.77	α-pinene	$C_{10}H_{16}$	136	933	937	1.65
2	5.52	β-myrcene	$C_{10}H_{16}$	136	983	984	2.45
3	6.17	*D*-limonene	$C_{10}H_{16}$	136	1018		72.71
4	6.37	β-ocimene	$C_{10}H_{16}$	136	1037	1037	1.03
5	7.14	linalool	$C_{10}H_{18}O$	154	1086	1095	2.86
6	9.38	geraniol	$C_{10}H_{18}O$	154	1237	1264	0.96
7	11.8	caryophyllene	$C_{15}H_{24}$	204	1419	1423	8.1
8	12.23	humulene	$C_{15}H_{24}$	204	1451	1436	0.51
9	12.57	germacrene D	$C_{15}H_{24}$	204	1477	1482	0.93
10	12.75	β-cyclogermacrane	$C_{15}H_{24}$	204	1492	1507	0.93
11	13.03	cadina-1(10),4-diene	$C_{15}H_{24}$	204	1469		0.35
12	13.36	elemol	$C_{15}H_{26}O$	222	1537	1550	0.25
13	13.91	(-)-globulol	$C_{15}H_{26}O$	222	1580	1580	0.24
14	14.05	β-eudesmol	$C_{15}H_{26}O$	222	1636	1639	0.2
15	14.52	γ-eudesmol	$C_{15}H_{26}O$	222	1618	1617	0.36
16	14.62	T-muurolol	$C_{15}H_{26}O$	222	1632	1644	0.3
17	14.68	agaruspirol	$C_{15}H_{26}O$	222	1631	1643	0.2
18	14.78	β-eudesmol	$C_{15}H_{26}O$	222	1636	1639	1.73
19	16.44	(E)-Farnesyl acetate	$C_{17}H_{28}O_2$	264	1817	1854	0.26
total							96.02

Retention indices [a]-calculated retention index, Retention indices [b]-retention index reported from previous reports.

Table S5. Chemical composition of *Tetradium fraxinifolium* leaf essential oil as shown by GC-MS analysis.

Peak number	Retention time (min)	Compound	Formula	Molecular Weight	Retention indices [a]	Retention indices [b]	Areas (%)
1	5.21	α-pinene	$C_{10}H_{16}$	136	933	937	18.51
2	5.44	camphene	$C_{10}H_{16}$	136	946		0.1
3	5.84	(-)-β-pinene	$C_{10}H_{16}$	136	943		0.22
4	5.96	β-myrcene	$C_{10}H_{16}$	136	983	984	13.82
5	6.58	*D*-limonene	$C_{10}H_{16}$	136	1018		0.38
6	6.64	*trans*-β-ocimene	$C_{10}H_{16}$	136	1038	1048	0.94
7	6.8	β-ocimene	$C_{10}H_{16}$	136	1037	1037	0.42
8	7.44	2-nonanone	$C_9H_{18}O$	142	1071	1092	3.29
9	7.55	2-nonanol	$C_9H_{20}O$	144	1089	1098	0.65
10	9.16	octyl acetate	$C_{10}H_{20}O_2$	172	1193	1189	0.13
11	10.34	2-undecanone	$C_{11}H_{22}O$	170	1273	1294	1.05
12	11.06	δ-elemene	$C_{15}H_{24}$	204	1337	1433	0.61
13	11.22	α-cubebene	$C_{15}H_{24}$	204	1351	1345	0.1
14	11.61	copaene	$C_{15}H_{24}$	204	1376	1362	0.54
15	11.78	β-elemen	$C_{15}H_{24}$	204	1388	1389	1.35
16	12.07	cyperene	$C_{15}H_{24}$	204	1398	1414	0.15
17	12.22	caryophyllene	$C_{15}H_{24}$	204	1419	1423	3.57
18	12.32	β-cubebene	$C_{15}H_{24}$	204	1385	1383	0.42
19	12.52	isogermacrene D	$C_{15}H_{24}$	204	1437	1451	0.1
20	12.65	humulene	$C_{15}H_{24}$	204	1451	1457	0.4
21	12.75	calarene	$C_{15}H_{24}$	204	1430	1423	0.2
22	12.89	γ-muurolene	$C_{15}H_{24}$	204	1472	1435	0.54
23	12.99	germacrene D	$C_{15}H_{24}$	204	1477	1482	11.12
24	13.18	β-cyclogermacrane	$C_{15}H_{24}$	204	1492	1507	2.75
25	13.27	β-cadinene	$C_{15}H_{24}$	204	1522	1518	0.13
26	13.37	γ-cadinene	$C_{15}H_{24}$	204	1507	1513	0.33
27	13.46	cadina-1(10),4-diene	$C_{15}H_{24}$	204	1469	1518	1.68
28	13.84	elemol	$C_{15}H_{26}O$	222	1537	1550	21.64
29	14.39	(-)-globulol	$C_{15}H_{26}O$	222	1530	1580	0.49
30	14.5	viridiflorol	$C_{15}H_{26}O$	222	1582	1609	0.19
31	14.84	γ-eudesmol	$C_{15}H_{26}O$	222	1618	1617	0.42
32	14.89	cubenol	$C_{15}H_{26}O$	222	1620	1607	0.1

33	15.03	T-muurolol	$C_{15}H_{26}O$	222	1632	1660	0.81
34	15.18	*β*-eudesmol	$C_{15}H_{26}O$	222	1636	1639	6.29
35	17.56	m-camphorene	$C_{20}H_{32}$	272	1944		0.1
36	18.51	phytol	$C_{20}H_{40}O$	296	2102		0.66
total							94.2

Retention indices [a]-calculated retention index, Retention indices [b]-retention index reported from previous reports.

Table S6. Chemical composition of *Tetradium fraxinifolium* fruit essential oil as shown by GC-MS analysis.

Peak number	Retention time (min)	Compound	Formula	Molecular Weight	Retention indices [a]	Retention indices [b]	Areas (%)
1	4.19	styrene	C_8H_8	104	878		0.16
2	4.78	cyclofenchene	$C_{10}H_{16}$	136	886	882	0.51
3	5.54	*β*-myrcene	$C_{10}H_{16}$	136	983	984	39.83
4	5.7	octanal	$C_8H_{16}O$	128	982	1004	0.29
5	5.82	hexyl acetate	$C_8H_{16}O_2$	144	995	996	0.25
6	6.14	*D*-limonene	$C_{10}H_{16}$	136	1018		9.58
7	6.21	*trans*-*β*-ocimene	$C_{10}H_{16}$	136	1038	1048	0.31
8	6.25	1-methylhexyl acetate	$C_9H_{18}O_2$	158	1022	1043	0.25
9	6.37	*β*-ocimene	$C_{10}H_{16}$	136	1037	1048	0.13
10	6.47	2-nonanone	$C_{10}H_{18}O$	142	1071	1092	0.23
11	6.68	1-octanol	$C_8H_{18}O$	130	1057	1061	1.09
12	7.04	2-nonanone	$C_9H_{18}O$	142	1071	1092	27.86
13	7.15	linalool	$C_{10}H_{18}O$	154	1086	1103	7.95
14	8.69	decanal	$C_{10}H_{20}O$	156	1185	1209	0.66
15	9	citronellol	$C_{10}H_{20}O$	156	1211	1128	0.12
16	9.38	geraniol	$C_{10}H_{18}O$	154	1237	1264	0.59
17	9.64	citral	$C_{10}H_{16}O$	152	1241	1239	0.22
18	9.93	2-undecanone	$C_{11}H_{22}O$	170	1273	1294	1.27
19	11.12	geranyl acetate	$C_{12}H_{20}O_2$	196	1361	1396	1.83
20	11.46	decyl acetate	$C_{12}H_{24}O_2$	200	1393	1409	0.36
21	11.91	vinyl caprylate	$C_{10}H_{18}O_2$	170	1173		0.15
22	12.57	*β*-copaene	$C_{15}H_{24}$	204	1426	1436	0.65
23	12.75	*γ*-elemene	$C_{15}H_{24}$	222	1432	1574	0.12
24	13.36	elemol	$C_{15}H_{26}O$	222	1537	1557	0.3
25	14	1-dodecanol	$C_{12}H_{26}O$	186	1462	1478	0.12
26	17.29	m-camphorene	$C_{20}H_{32}$	272	1944	1994	0.13
total							94.96

Table S7. Chemical composition of *Zanthoxylum armatum* leaf essential oil as shown by GC-MS analysis.

Peak number	Retention time (min)	Compound	Formula	Molecular Weight	Retention indices [a]	Retention indices [b]	Areas (%)
1	4.85	α-pinene	$C_{10}H_{16}$	136	933	927	1.03
2	5.4	sabinen	$C_{10}H_{16}$	136	967	946	5.41
3	5.47	(-)-β-pinene	$C_{10}H_{16}$	136	943		0.38
4	5.59	β-myrcene	$C_{10}H_{16}$	136	983	984	1.74
5	5.85	α-phellandrene	$C_{10}H_{16}$	136	998	997	0.76
6	6.02	terpinolene	$C_{10}H_{16}$	136	1079	1010	0.13
7	6.14	*o*-cymene	$C_{10}H_{14}$	134	1025	1015	0.18
8	6.21	*D*-limonene	$C_{10}H_{16}$	136	1018		10.7
9	6.27	eucalyptol	$C_{10}H_{18}O$	154	1022	1022	29.65
10	6.44	*cis*-β-ocimene	$C_{10}H_{16}$	136	1028	1035	0.64
11	6.65	γ-terpinene	$C_{10}H_{16}$	136	1050	1060	0.45
12	7.1	terpinolene	$C_{10}H_{16}$	136	1079	1089	0.16
13	7.21	linalool	$C_{10}H_{18}O$	154	1086	1095	0.67
14	8.28	δ-terpineol	$C_{10}H_{18}O$	154	1146	1134	0.2
15	8.44	terpinen-4-ol	$C_{10}H_{18}O$	154	1164	1167	0.82
16	8.56	myrtanal	$C_{10}H_{16}O$	152	1126	1180	0.2
17	8.62	α-terpineol	$C_{10}H_{18}O$	154	1175	1183	0.47
18	8.72	myrtenol	$C_{10}H_{16}O$	152	1181	1280	0.6
19	9.63	(-)-*cis*-myrtanol	$C_{10}H_{18}O$	154	1180		0.24
20	9.99	2-undecanone	$C_{11}H_{22}O$	170	1273	1610	0.34
21	10.5	myrtenyl acetate	$C_{12}H_{18}O_2$	194	1305	1326	0.41
22	11.29	(E)-methyl cinnamate	$C_{10}H_{10}O_2$	162	1364	1397	0.88
23	11.43	(-)-β-elemene	$C_{15}H_{24}$	204	1388	1389	4.07
24	11.86	caryophyllene	$C_{15}H_{24}$	204	1419	1423	2.24
25	12.3	humulene	$C_{15}H_{24}$	204	1451	1436	0.67
26	12.63	germacrene D	$C_{15}H_{24}$	204	1477	1482	1.91
27	12.81	bicyclogermacren	$C_{15}H_{24}$	204	1492	1486	0.55
28	13.09	(-)-β-cadinene	$C_{15}H_{24}$	204	1522	1518	0.26
29	13.5	D-nerolidol	$C_{15}H_{26}O$	222	1527	1571	0.22
30	13.59	isoaromadendrene epoxide	$C_{15}H_{24}O$	220	1590	1807	0.32
31	13.99	caryophyllene oxide	$C_{15}H_{24}O$	220	1574	1593	2.71
32	14.11	d-viridiflorol	$C_{15}H_{26}O$	222	1582	1609	0.16

33	14.34	humulene oxide II	$C_{15}H_{24}O$	220	1599	1579	1.63
34	14.69	T-muurolol	$C_{15}H_{26}O$	222	1632	1660	0.36
35	14.84	α-cadinol	$C_{15}H_{26}O$	222	1642	1653	0.68
36	15.74	myristic acid	$C_{14}H_{28}O_2$	228	1752	1769	0.31
37	17.3	palmitic acid	$C_{16}H_{32}O_2$	256	1954	1975	1.86
38	18.26	phytol	$C_{20}H_{40}O$	296	2102	2145	4.34
39	18.4	linolenic acid	$C_{18}H_{30}O_2$	278	2115	2134	6.22
total							84.57

Retention indices [a]-calculated retention index, Retention indices [b]-retention index reported from previous reports.

Table S8. Chemical composition of *Ruta graveolens* leaf essential oil as shown by GC-MS analysis.

Peak number	Retention time (min)	Compound	Formula	Molecular Weight	Retention indices [a]	Retention indices [b]	Areas (%)
1	3.21	butyl acetate	$C_6H_{12}O_2$	116	796	1063	0.77
2	3.55	diacetone alcohol	$C_6H_{12}O_2$	116	816	838	0.38
3	6.21	*trans*-*β*-ocimene	$C_{10}H_{16}$	136	1038	1048	0.2
4	7.15	linalool	$C_{10}H_{18}O$	154	1086	1103	0.23
5	10.63	*δ*-elemene	$C_{15}H_{24}$	204	1337	1335	4.44
6	10.8	*α*-copaene	$C_{15}H_{24}$	204	1376	1362	0.12
7	11.19	ylangene	$C_{15}H_{24}$	204	1370	1365	0.15
8	11.37	*β*-elemene	$C_{15}H_{24}$	204	1388	1389	3.74
9	11.79	caryophyllene	$C_{15}H_{24}$	204	1419	1423	2.85
10	11.9	isogermacrene D	$C_{15}H_{24}$	204	1437	1442	1.6
11	12.04	aromandendrene	$C_{15}H_{24}$	204	1447	1440	0.4
12	12.1	*β*-copaene	$C_{15}H_{24}$	204	1426	1422	0.14
13	12.23	humulene	$C_{15}H_{24}$	204	1451	1436	1.14
14	12.33	*γ*-muurolene	$C_{15}H_{24}$	204	1472	1435	0.31
15	12.47	*γ*-muurolene	$C_{15}H_{24}$	204	1472		2.32
16	12.52	*α*-muurolene	$C_{15}H_{24}$	204	1440		1.76
17	12.57	germacrene D	$C_{15}H_{24}$	204	1477	1482	17.57
18	12.65	*β*-patchoulene	$C_{15}H_{24}$	204	1377	1374	0.61
19	12.75	*α*-muurolene	$C_{15}H_{24}$	204	1440		8.17
20	12.85	cadina-1(10),4-diene	$C_{15}H_{24}$	204	1469		1.4
21	12.95	*γ*-cadinene	$C_{15}H_{24}$	204	1507	1513	1.46
22	13.03	(-)-*β*-cadinene	$C_{15}H_{24}$	204	1522	1518	5.65
23	13.23	*α*-muurolene	$C_{15}H_{24}$	204	1440	1365	0.54
24	13.32	*α*-calacorene	$C_{15}H_{20}$	200	1531	1524	0.48
25	13.44	E-nerolidol	$C_{15}H_{26}O$	222	1549	1571	1.27
26	13.55	aromandendrene	$C_{15}H_{24}$	204	1447	1558	1.47
27	13.69	*γ*-eudesmol	$C_{15}H_{26}O$	222	1618	1617	0.55
28	13.81	spathulenol	$C_{15}H_{24}O$	220	1568	1577	0.49
29	13.91	(-)-globulol	$C_{15}H_{26}O$	222	1530	1580	1.99
30	14.04	viridiflorol	$C_{15}H_{26}O$	222	1582	1590	1.28
31	14.16	rosifoliol	$C_{15}H_{26}O$	222	1598	1612	0.44
32	14.31	cubenol	$C_{15}H_{26}O$	222	1620	1607	0.53
33	14.42	junenol	$C_{15}H_{26}O$	222	1613	1629	1.47
34	14.46	isospathulenol	$C_{15}H_{24}O$	220	1628	1652	2.14

35	14.53	cubenol	$C_{15}H_{26}O$	222	1620	1607	0.52
36	14.62	T-muurolol	$C_{15}H_{26}O$	222	1632	1660	4.66
37	14.78	α-cadinol	$C_{15}H_{26}O$	222	1642	1653	7.62
38	14.99	isoaromadendrene epoxide	$C_{15}H_{24}O$	220	1590	1807	0.97
39	16.45	nerolidyl acetate	$C_{17}H_{28}O_2$	264	1754	1687	0.74
40	17.27	n-hexadecanoic acid	$C_{16}H_{32}O_2$	256	1954		4.59
41	18.23	phytol	$C_{20}H_{40}O$	296	2102	2145	0.99
42	18.37	linolic acid	$C_{18}H_{32}O_2$	352	2113		5.17
total							93.32

Retention indices [a]-calculated retention index, Retention indices [b]-retention index reported from previous reports.

Table S9. Chemical composition of *Ruta graveolens* fruit essential oil as shown by GC-MS analysis.

Peak number	Retention time (min)	Compound	Formula	Molecular Weight	Retention indices [a]	Retention indices [b]	Areas (%)
1	6.76	2-nonanone	$C_9H_{18}O$	142	1071	1092	21.6
2	6.86	2-nonanol	$C_9H_{20}O$	144	1086	1098	0.81
3	6.93	nonanal	$C_9H_{18}O$	142	1083	1112	0.16
4	7.57	Geijerene	$C_{12}H_{18}$	162	1138	1147	2.03
5	8.23	2-decanone	$C_{10}H_{20}O$	156	1172	1193	1.78
6	9.72	2-undecanone	$C_{11}H_{22}O$	170	1273	1294	62.98
7	9.75	2-undecanol	$C_{11}H_{24}O$	172	1287	1285	1.62
8	10.62	2-dodecanone	$C_{12}H_{24}O$	184	1377	1395	0.58
9	10.88	α-copaene	$C_{15}H_{24}$	204	1376	1362	0.36
10	11.07	(-)-β-elemene	$C_{15}H_{24}$	184	1388	1395	0.59
11	11.49	caryophyllene	$C_{15}H_{24}$	204	1419	1423	1.32
12	11.92	humulene	$C_{15}H_{24}$	204	1451	1457	0.19
13	12.28	2-tridecanone	$C_{13}H_{26}O$	198	1477	1495	1.53
14	13.05	elemol	$C_{15}H_{26}O$	222	1537	1550	0.29
15	14.31	T-muurolol	$C_{15}H_{26}O$	222	1632	1660	0.15
16	14.47	α-cadinol	$C_{15}H_{26}O$	222	1642	1653	0.2
17	18.35	isopimaral	$C_{20}H_{30}O$	286	2114		0.16
total							96.35

Retention indices [a]-calculated retention index, Retention indices [b]-retention index reported from previous reports.

Table S10. Chemical composition of *Citrus medica* leaf essential oil as shown by GC-MS analysis.

Peak number	Retention time (min)	Compound	Formula	Molecular Weight	Retention indices [a]	Retention indices [b]	Areas (%)
1	5.53	β-myrcene	$C_{10}H_{16}$	136	983	984	10.82
2	5.78	α-phellandrene	$C_{10}H_{16}$	136	998	997	0.11
3	6.14	*D*-limonene	$C_{10}H_{16}$	136	1018		0.9
4	6.22	*trans*-β-ocimene	$C_{10}H_{16}$	136	1038	1048	0.16
5	6.38	β-ocimene	$C_{10}H_{16}$	136	1037	1037	5.23
6	7.15	linalool	$C_{10}H_{18}O$	154	1086	1103	1.08
7	9.04	*cis*-geraniol	$C_{10}H_{18}O$	154	1213	1230	0.11
8	9.24	neral	$C_{10}H_{16}O$	152	1218	1241	0.17
9	9.38	geraniol	$C_{10}H_{18}O$	154	1237	1264	0.12
10	9.64	α-citral	$C_{10}H_{16}O$	152	1249	1270	0.23
11	10.63	δ-elemene	$C_{15}H_{24}$	204	1337	1340	1.95
12	11.28	(-)-β-elemene	$C_{15}H_{24}$	204	1388	1389	1.02
13	11.39	(-)-β-elemene	$C_{15}H_{24}$	204	1388		18.86
14	11.81	caryophyllene	$C_{15}H_{24}$	204	1419	1423	12.91
15	11.9	germacrene B	$C_{15}H_{24}$	204	1550	1558	6.1
16	12.05	β-eudesmene	$C_{15}H_{24}$	204	1482	1493	0.22
17	12.1	aromandendrene	$C_{15}H_{24}$	204	1447	1440	0.39
18	12.23	humulene	$C_{15}H_{24}$	204	1451	1436	3.23
19	12.47	eremophilene	$C_{15}H_{24}$	204	1486	1500	1
20	12.57	germacrene D	$C_{15}H_{24}$	204	1477	1482	9.37
21	12.65	β-eudesmene	$C_{15}H_{24}$	204	1482	1493	0.62
22	12.76	β-cyclogermacrane	$C_{15}H_{24}$	204	1492	1500	2.46
23	12.88	(-)-β-elemene	$C_{15}H_{24}$	204	1388	1389	0.56
24	12.95	γ-cadinene	$C_{15}H_{24}$	204	1507	1513	0.2
25	13.03	(-)-β-cadinene	$C_{15}H_{24}$	204	1522	1518	0.48
26	13.1	cembrene	$C_{20}H_{32}$	222	1934	1648	0.19
27	13.23	costol	$C_{15}H_{24}O$	220	1769	1774	0.48
28	13.36	elemol	$C_{15}H_{26}O$	222	1537	1557	0.57
29	13.44	E-nerolidol	$C_{15}H_{26}O$	222	1549	1571	1.48
30	13.77	cubebol	$C_{15}H_{26}O$	222	1510	1515	0.11
31	13.81	spathulenol	$C_{15}H_{24}O$	220	1568	1577	0.12
32	13.92	caryophyllene oxide	$C_{15}H_{24}O$	220	1574	1561	0.39
33	14.31	neointermedeol	$C_{15}H_{26}O$	222	1601	1662	0.12
34	14.38	cubebol	$C_{15}H_{26}O$	222	1510	1515	0.22

35	14.46	isospathulenol	$C_{15}H_{24}O$	220	1628	1640	0.39
36	14.63	T-cadinol	$C_{15}H_{26}O$	222	1627	1644	0.53
37	14.78	α-cadinol	$C_{15}H_{26}O$	222	1642	1653	0.45
38	14.82	neointermedeol	$C_{15}H_{26}O$	222	1601	1662	0.45
39	15.43	isoaromadendrene epoxide	$C_{15}H_{24}O$	220	1590	1807	0.11
40	17.27	n-hexadecanoic acid	$C_{16}H_{32}O_2$	256	1954	1975	0.55
41	18.22	phytol	$C_{20}H_{40}O$	296	2102	2145	5.91
42	18.32	linolic acid	$C_{18}H_{32}O_2$	280	2113	2113	0.12
43	18.36	linolenic acid	$C_{18}H_{32}O_2$	278	2115	2134	0.59
44	18.45	β-monolinolein	$C_{21}H_{38}O_4$	354	2713		0.15
45	20.17	n-pentacosane	$C_{25}H_{52}$	352	2506	394	0.69
total							91.92

Retention indices [a]-calculated retention index, Retention indices [b]-retention index reported from previous reports.

Table S11. Chemical composition of *Citrus medica* fruit essential oil as shown by GC-MS analysis.

Peak number	Retention time (min)	Compound	Formula	Molecular Weight	Retention indices [a]	Retention indices [b]	Areas (%)
1	4.42	α-thujene	$C_{10}H_{16}$	136	925	929	0.74
2	4.53	α-pinene	$C_{10}H_{16}$	136	933	1052	2.02
3	5.15	(-)-β-pinene	$C_{10}H_{16}$	136	943		2.32
4	5.27	β-myrcene	$C_{10}H_{16}$	136	983	984	1.4
5	5.82	*p*-cymene	$C_{10}H_{14}$	134	1014	1028	6.95
6	5.92	D-limonene	$C_{10}H_{16}$	136	1018		39.77
7	6.34	γ-terpinene	$C_{10}H_{16}$	136	1050	1060	27.81
8	6.76	α-terpinolen	$C_{10}H_{16}$	136	1079	1277	1.93
9	6.9	(+)-3-carene	$C_{10}H_{16}$	136	1006	1010	2.12
10	8.11	*trans*-4-thujanol	$C_{10}H_{18}O$	154	1062	1079	1.88
11	8.3	α-terpineol	$C_{10}H_{18}O$	154	1175	1183	2.43
12	8.98	carveol	$C_{10}H_{16}$	152	1207	1200	2.01
13	9.38	citral	$C_{10}H_{16}O$	152	1241	1216	2.67
14	10.61	*p*-mentha-1(7),8-diene	$C_{10}H_{16}$	136	993	1006	1.57
15	10.86	(-)-β-pinene	$C_{10}H_{16}$	136	943		0.97
16	12.27	β-copaene	$C_{15}H_{24}$	204	1426	1422	0.65
17	12.51	β-bisabolene	$C_{15}H_{24}$	204	1500	1509	0.42
total							97.66

Retention indices [a]-calculated retention index, Retention indices [b]-retention index reported from previous reports.

Table S12. Extraction yield of essential oils from twelve materials.

Plant	EO Extraction Yield (%)
Tetradium ruticarpum leaf	1.00
Tetradium ruticarpum fruit	2.65
Tetradium daniellii leaf	0.92
Tetradium daniellii fruit	1.40
Tetradium fraxinifolium leaf	0.35
Tetradium fraxinifolium fruit	3.75
Zanthoxylum armatum leaf	0.45
Zanthoxylum armatum fruit	4.00
Ruta graveolens leaf	0.11
Ruta graveolens fruit	0.82
Citrus medica leaf	0.10
Citrus medica fruit	1.12

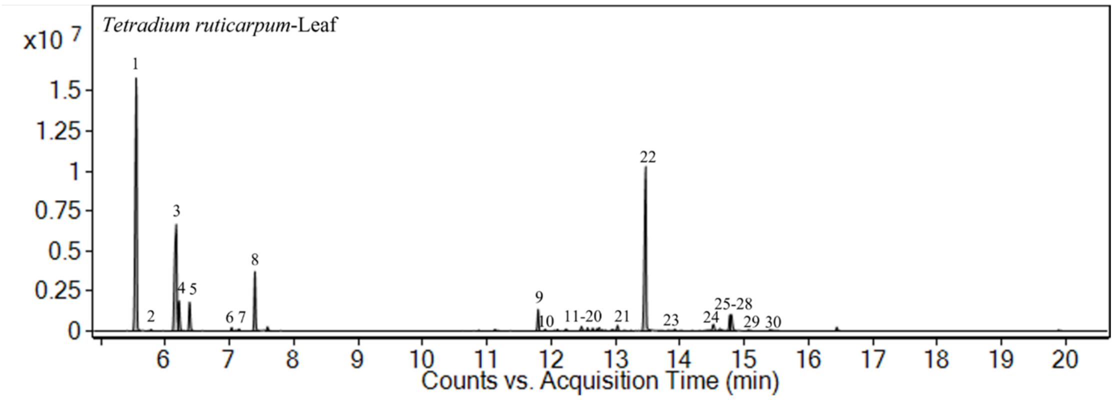

Figure S1. GC-MS chromatogram of *Tetradium ruticarpum* leaf essential oil. The corresponding peaks were marked with the represented substances as shown in Table S1.

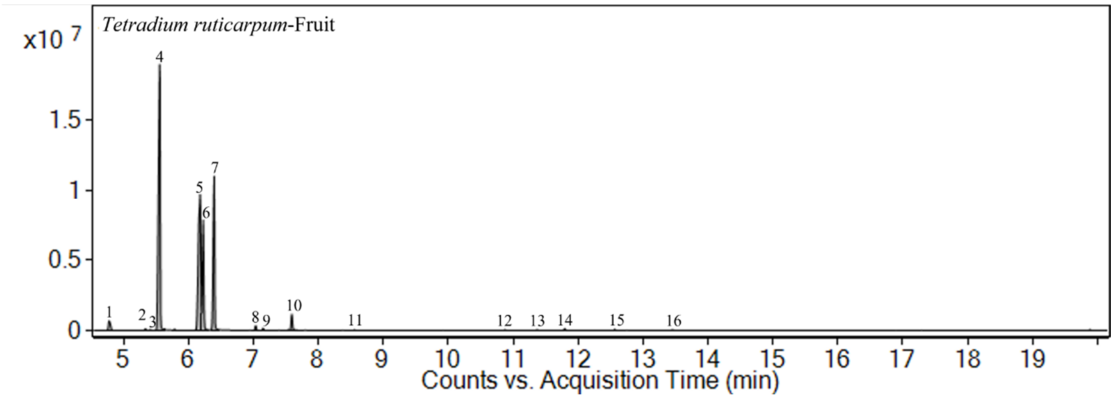

Figure S2. GC-MS chromatogram of *Tetradium ruticarpum* fruit essential oil. The corresponding peaks were marked with the represented substances as shown in Table S2.

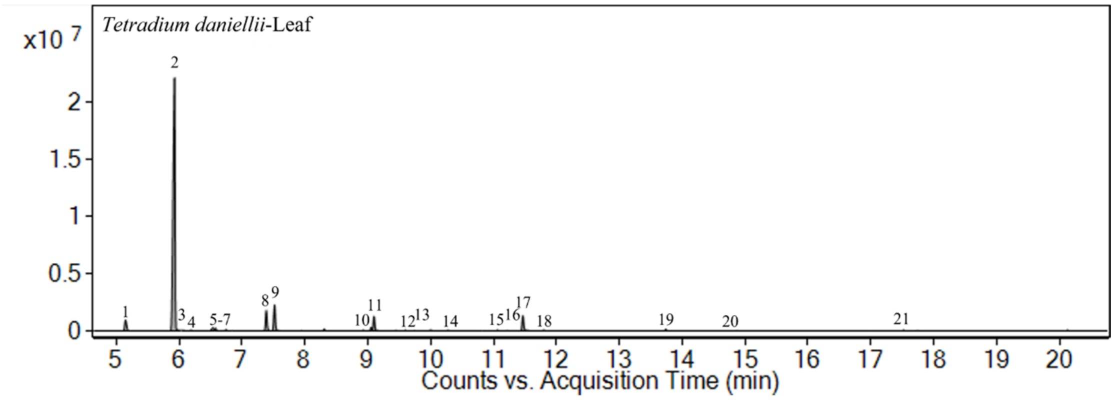

Figure S3. GC-MS chromatogram of *Tetradium daniellii* leaf essential oil. The corresponding peaks were marked with the represented substances as shown in Table S3.

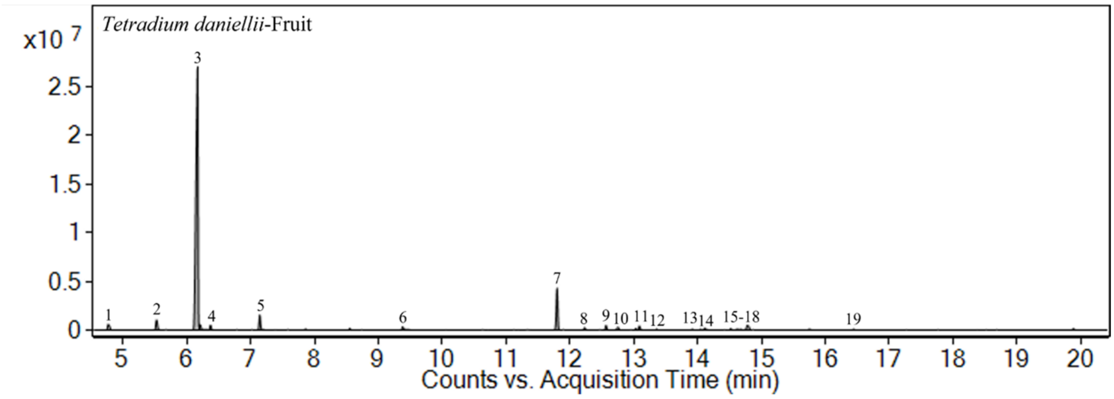

Figure S4. GC-MS chromatogram of *Tetradium daniellii* fruit essential oil. The corresponding peaks were marked with the represented substances as shown in Table S4.

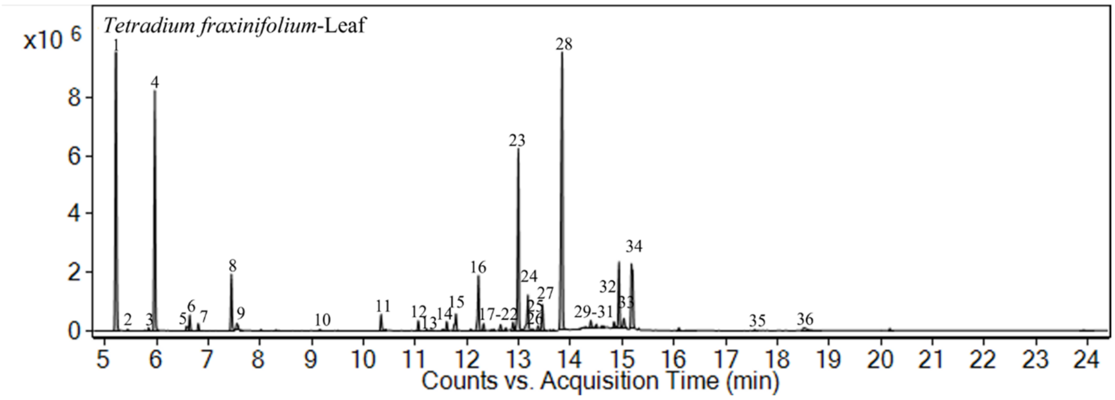

Figure S5. GC-MS chromatogram of *Tetradium fraxinifolium* leaf essential oil. The corresponding peaks were marked with the represented substances as shown in Table S5.

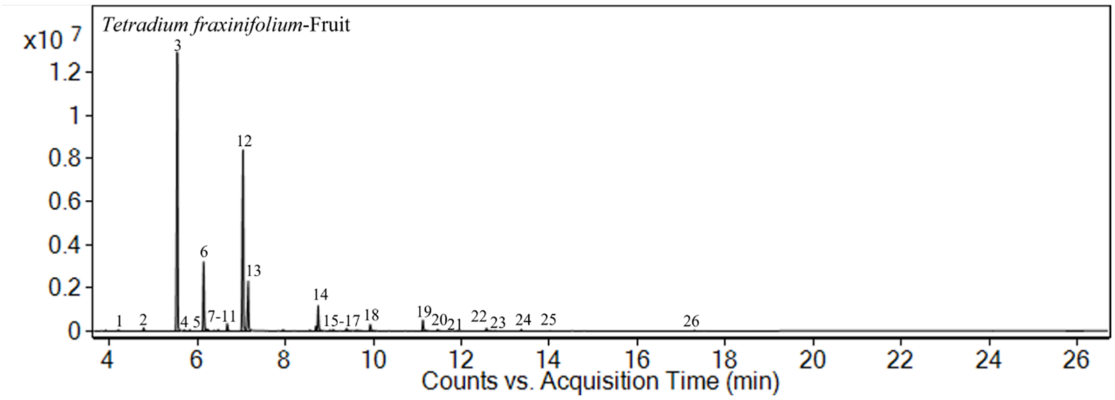

Figure S6. GC-MS chromatogram of *Tetradium fraxinifolium* fruit essential oil. The corresponding peaks were marked with the represented substances as shown in Table S6.

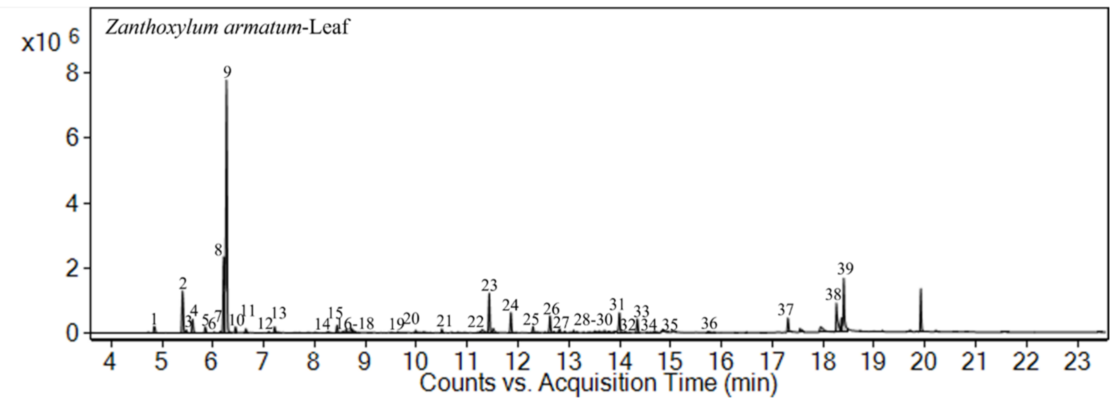

Figure S7. GC-MS chromatogram of *Zanthoxylum armatum* leaf essential oil. The corresponding peaks were marked with the represented substances as shown in Table S7.

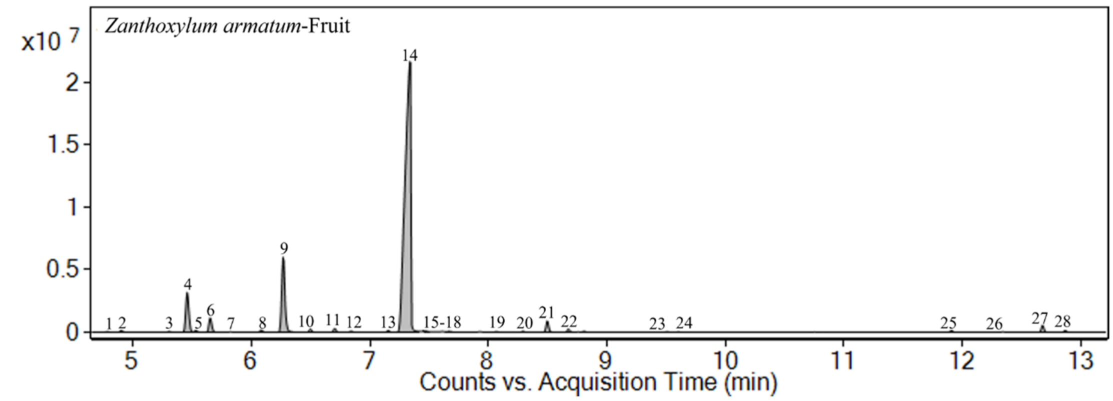

Figure S8. GC-MS chromatogram of *Zanthoxylum armatum* fruit essential oil. The corresponding peaks were marked with the represented substances as shown in Table 1.

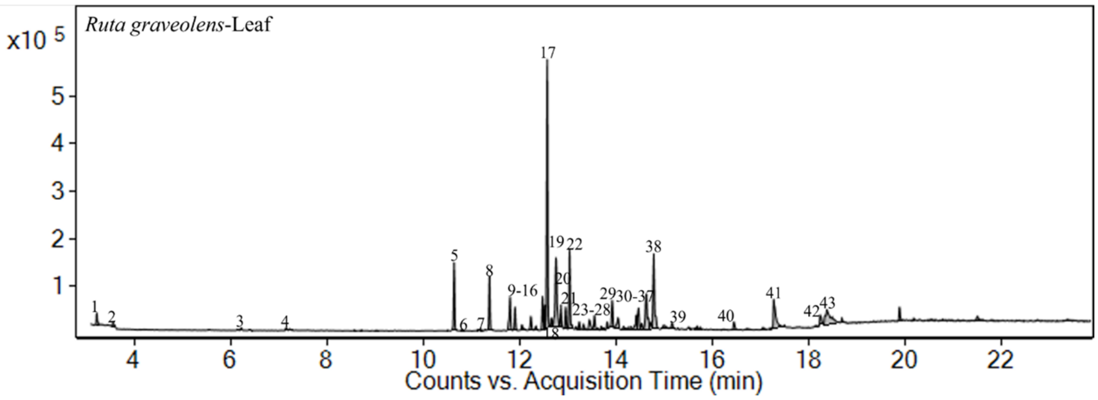

Figure S9. GC-MS chromatogram of *Ruta graveolens* leaf essential oil. The corresponding peaks were marked with the represented substances as shown in Table S8.

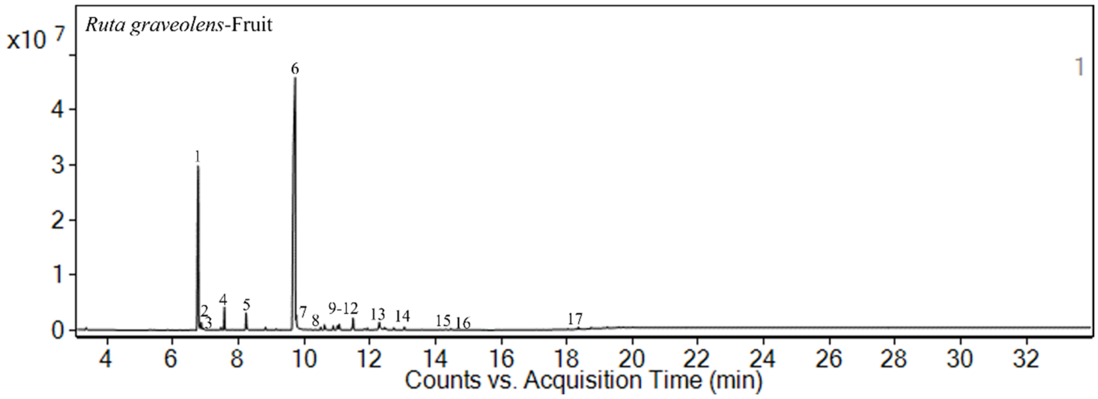

Figure S10. GC-MS chromatogram of *Ruta graveolens* fruit essential oil. The corresponding peaks were marked with the represented substances as shown in Table S9.

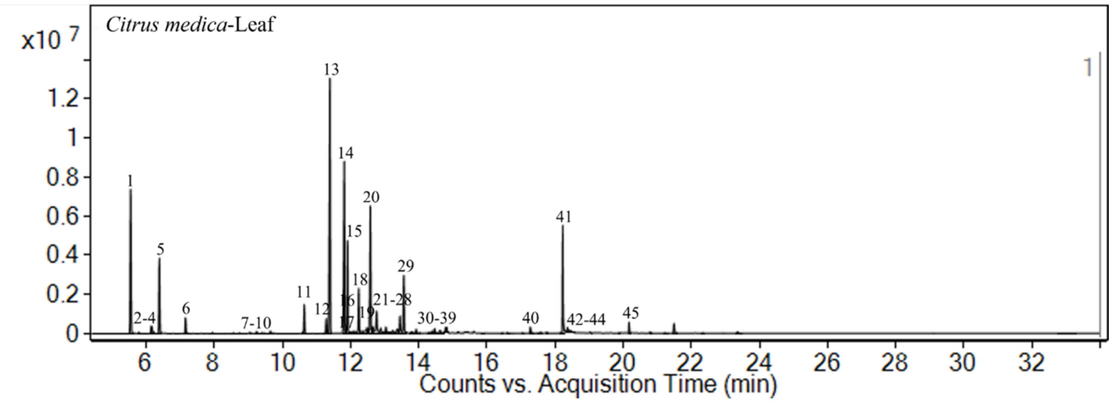

Figure S11. GC-MS chromatogram of *Citrus medica* leaf essential oil. The corresponding peaks were marked with the represented substances as shown in Table S10.

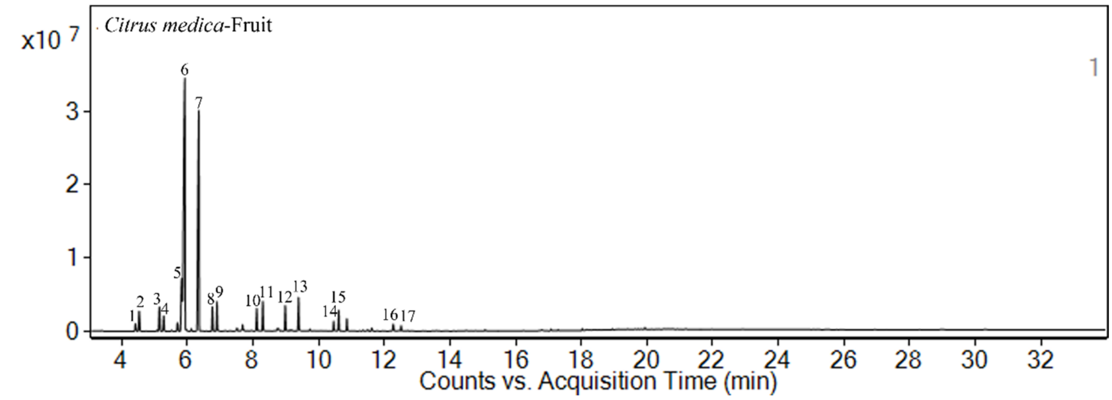

Figure S12. GC-MS chromatogram of *Citrus medica* fruit essential oil. The corresponding peaks were marked with the represented substances as shown in Table S11.

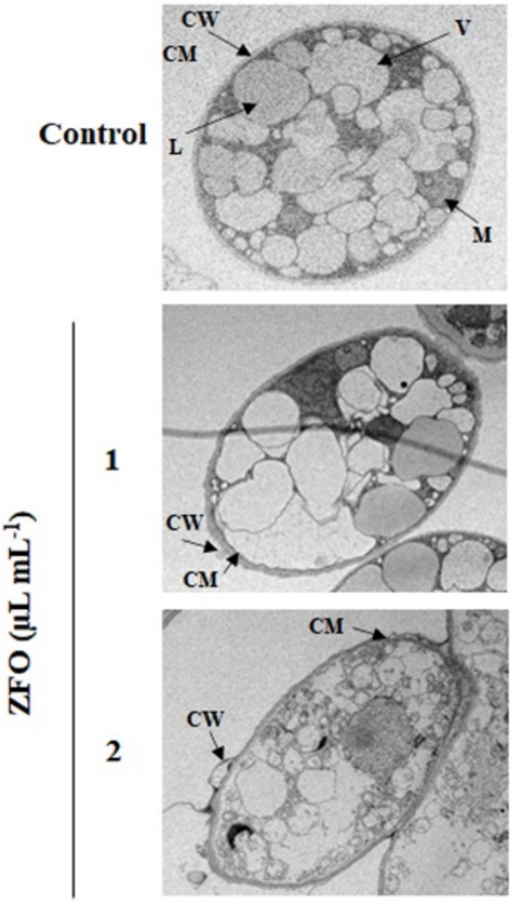

Figure S13. Transmission electron microscopy observations were examined to explore the effects of ZFO on the ultrastructure. CM: cell membrane; CW: cell wall; M: mitochondria; V: vacuole; and L: lipidosome.
